# Supplementary material for: Opposing Network Patterns of Integration–Segregation in Psychedelic and Sedated States of Consciousness
Source: bioRxiv. 2026 Feb 18:2026.02.17.706398. Preprint. [Version 1] doi: 10.64898/2026.02.17.706398 (PMC12934629; doi:10.64898/2026.02.17.706398)
Supplement: Supplement 1 [file NIHPP2026.02.17.706398v1-supplement-1.pdf]

bioRxiv preprint doi: https://doi.org/10.64898/2026.02.17.706398; this version posted February 18, 2026. The copyright holder for this preprint (which was not certified by peer review) is the author/funder, who has granted bioRxiv a license to display the preprint in perpetuity. It is made available under aCC-BY 4.0 International license.

Supplementary Table S1. Global within-network results

Metric	Scale	State	Baseline mean	State mean	Cohen's d	SE	t	p_unc	p_FDR
FC	Global mean	LSD	0.105	0.124	0.551	0.009	2.134	0.051	0.092
FC	Global mean	PSIL	0.537	0.641	0.493	0.080	1.305	0.240	0.308
FC	Global mean	KTM	0.226	0.260	0.851	0.011	2.948	0.013	0.030
FC	Global mean	N2O	0.351	0.391	0.277	0.037	1.072	0.302	0.340
FC	Global mean	Sleep	0.422	0.410	-0.132	0.016	-0.755	0.456	0.456
FC	Global mean	Sleep	0.424	0.365	-0.602	0.018	-3.240	0.003	0.009
FC	Global mean	PPF1.9	0.376	0.313	-0.349	0.048	-1.306	0.214	0.308
FC	Global mean	PPF2.4	0.307	0.246	-0.713	0.017	-3.637	0.001	0.006
FC	Global mean	PPF2.7	0.412	0.183	-1.576	0.028	-8.187	0.000	0.000
Efficiency	Global mean	LSD	0.626	0.706	1.371	0.015	5.311	0.000	0.001
Efficiency	Global mean	PSIL	0.752	0.792	0.502	0.030	1.327	0.233	0.349
Efficiency	Global mean	KTM	0.705	0.726	0.505	0.012	1.750	0.108	0.199
Efficiency	Global mean	N2O	0.706	0.711	0.142	0.009	0.550	0.591	0.665
Efficiency	Global mean	Sleep	0.687	0.678	-0.286	0.005	-1.641	0.111	0.199
Efficiency	Global mean	Sleep	0.687	0.652	-0.749	0.009	-4.032	0.000	0.002
Efficiency	Global mean	PPF1.9	0.659	0.613	-0.922	0.013	-3.450	0.004	0.013
Efficiency	Global mean	PPF2.4	0.670	0.665	-0.067	0.014	-0.342	0.735	0.735
Efficiency	Global mean	PPF2.7	0.649	0.638	-0.129	0.017	-0.670	0.509	0.654
Complexity	Global mean	LSD	0.152	0.156	0.454	0.002	1.758	0.101	0.181
Complexity	Global mean	PSIL	0.173	0.177	0.409	0.004	1.082	0.321	0.454
Complexity	Global mean	KTM	0.197	0.199	0.280	0.002	0.970	0.353	0.454
Complexity	Global mean	N2O	0.191	0.189	-0.216	0.003	-0.836	0.417	0.469
Complexity	Global mean	Sleep	0.189	0.189	0.006	0.001	0.034	0.973	0.973
Complexity	Global mean	Sleep	0.189	0.185	-0.566	0.001	-3.046	0.005	0.011
Complexity	Global mean	PPF1.9	0.191	0.182	-1.076	0.002	-4.028	0.001	0.004
Complexity	Global mean	PPF2.4	0.112	0.100	-1.392	0.002	-7.100	0.000	0.000
Complexity	Global mean	PPF2.7	0.152	0.138	-1.666	0.002	-8.657	0.000	0.000

Abbreviations: FC: functional connectivity; LSD: lysergic acid diethylamide; PSIL: psilocybin; KTM: ketamine; N_2O: nitrous oxide; PPF1.9 / PPF2.4 / PPF2.7: propofol (effect-site concentrations: 1.9 / 2.4 / 2.7 $\mu g \cdot mL^{-1}$).

bioRxiv preprint doi: https://doi.org/10.64898/2026.02.17.706398; this version posted February 18, 2026. The copyright holder for this preprint (which was not certified by peer review) is the author/funder, who has granted bioRxiv a license to display the preprint in perpetuity. It is made available under aCC-BY 4.0 International license.

Supplementary Table S2. Global between-network results

Metric	Scale	State	Baseline mean	State mean	Cohen's d	SE	t	p_unc	p_FDR
FC	Global between-network mean	LSD	0.067	0.090	0.658	0.009	2.547	0.023	0.042
FC	Global between-network mean	PSIL	0.518	0.618	0.450	0.084	1.190	0.279	0.359
FC	Global between-network mean	KTM	0.201	0.234	0.791	0.012	2.740	0.019	0.042
FC	Global between-network mean	N2O	0.314	0.349	0.247	0.037	0.957	0.355	0.399
FC	Global between-network mean	Sleep N1	0.388	0.377	-0.116	0.016	-0.664	0.511	0.511
FC	Global between-network mean	Sleep N2	0.389	0.332	-0.593	0.018	-3.192	0.003	0.010
FC	Global between-network mean	PPF1.9	0.346	0.273	-0.426	0.046	-1.595	0.135	0.202
FC	Global between-network mean	PPF2.4	0.282	0.221	-0.725	0.017	-3.699	0.001	0.005
FC	Global between-network mean	PPF2.7	0.372	0.150	-1.588	0.027	-8.251	0.000	0.000
Efficiency	Global between-network mean	LSD	0.649	0.695	1.581	0.007	6.124	0.000	0.000
Efficiency	Global between-network mean	PSIL	0.754	0.782	0.362	0.029	0.959	0.375	0.422
Efficiency	Global between-network mean	KTM	0.706	0.723	0.633	0.008	2.193	0.051	0.114
Efficiency	Global between-network mean	N2O	0.709	0.708	-0.020	0.006	-0.078	0.939	0.939
Efficiency	Global between-network mean	Sleep N1	0.697	0.692	-0.270	0.003	-1.552	0.130	0.171
Efficiency	Global between-network mean	Sleep N2	0.696	0.672	-0.813	0.005	-4.376	0.000	0.000
Efficiency	Global between-network mean	PPF1.9	0.663	0.610	-1.666	0.008	-6.232	0.000	0.000
Efficiency	Global between-network mean	PPF2.4	0.699	0.684	-0.326	0.009	-1.664	0.109	0.171
Efficiency	Global between-network mean	PPF2.7	0.673	0.651	-0.298	0.014	-1.550	0.133	0.171
Complexity	Global between-network mean	LSD	0.244	0.249	0.630	0.002	2.438	0.029	0.052
Complexity	Global between-network mean	PSIL	0.269	0.275	0.454	0.005	1.200	0.275	0.413
Complexity	Global between-network mean	KTM	0.305	0.306	0.198	0.003	0.684	0.508	0.653
Complexity	Global between-network mean	N2O	0.288	0.286	-0.103	0.004	-0.398	0.697	0.784
Complexity	Global between-network mean	Sleep N1	0.286	0.286	-0.035	0.001	-0.202	0.841	0.841
Complexity	Global between-network mean	Sleep N2	0.286	0.280	-0.630	0.002	-3.392	0.002	0.005
Complexity	Global between-network mean	PPF1.9	0.292	0.279	-1.049	0.003	-3.923	0.002	0.005
Complexity	Global between-network mean	PPF2.4	0.174	0.162	-1.446	0.002	-7.374	0.000	0.000
Complexity	Global between-network mean	PPF2.7	0.230	0.220	-1.055	0.002	-5.482	0.000	0.000

Abbreviations: FC: functional connectivity; LSD: lysergic acid diethylamide; PSIL: psilocybin; KTM: ketamine; N_2O: nitrous oxide; PPF1.9 / PPF2.4 / PPF2.7: propofol (effect-site concentrations: 1.9 / 2.4 / 2.7 $\mu g \cdot mL^{-1}$).

bioRxiv preprint doi: https://doi.org/10.64898/2026.02.17.706398; this version posted February 18, 2026. The copyright holder for this preprint (which was not certified by peer review) is the author/funder, who has granted bioRxiv a license to display the preprint in perpetuity. It is made available under aCC-BY 4.0 International license.

Supplementary Table S3. Global within-network results

Metric	Scale	State	Baseline mean	State mean	Cohen's d	SE	t	p_unc	p_FDR
FC	Global within-network mean	LSD	0.323	0.304	-0.597	0.008	-2.311	0.037	0.082
FC	Global within-network mean	PSIL	0.629	0.702	0.472	0.059	1.248	0.259	0.333
FC	Global within-network mean	KTM	0.362	0.365	0.083	0.009	0.286	0.780	0.780
FC	Global within-network mean	N2O	0.481	0.495	0.120	0.031	0.465	0.649	0.730
FC	Global within-network mean	Sleep N1	0.550	0.533	-0.233	0.012	-1.337	0.191	0.286
FC	Global within-network mean	Sleep N2	0.550	0.495	-0.744	0.014	-4.005	0.000	0.002
FC	Global within-network mean	PPF1.9	0.512	0.458	-0.382	0.037	-1.430	0.176	0.286
FC	Global within-network mean	PPF2.4	0.446	0.388	-0.748	0.015	-3.813	0.001	0.002
FC	Global within-network mean	PPF2.7	0.554	0.348	-1.465	0.027	-7.613	0.000	0.000
Efficiency	Global within-network mean	LSD	0.804	0.833	1.259	0.006	4.878	0.000	0.001
Efficiency	Global within-network mean	PSIL	0.864	0.891	0.718	0.014	1.899	0.106	0.137
Efficiency	Global within-network mean	KTM	0.824	0.832	0.452	0.006	1.567	0.145	0.163
Efficiency	Global within-network mean	N2O	0.825	0.819	-0.370	0.005	-1.433	0.174	0.174
Efficiency	Global within-network mean	Sleep N1	0.824	0.814	-0.426	0.004	-2.449	0.020	0.030
Efficiency	Global within-network mean	Sleep N2	0.823	0.793	-0.867	0.006	-4.670	0.000	0.000
Efficiency	Global within-network mean	PPF1.9	0.808	0.754	-1.901	0.008	-7.113	0.000	0.000
Efficiency	Global within-network mean	PPF2.4	0.819	0.798	-0.690	0.006	-3.521	0.002	0.004
Efficiency	Global within-network mean	PPF2.7	0.817	0.788	-0.530	0.010	-2.754	0.011	0.019
Complexity	Global within-network mean	LSD	0.248	0.252	0.417	0.002	1.615	0.129	0.232
Complexity	Global within-network mean	PSIL	0.264	0.267	0.367	0.004	0.971	0.369	0.494
Complexity	Global within-network mean	KTM	0.301	0.300	-0.121	0.002	-0.420	0.683	0.709
Complexity	Global within-network mean	N2O	0.286	0.283	-0.232	0.003	-0.898	0.384	0.494
Complexity	Global within-network mean	Sleep N1	0.283	0.283	-0.066	0.001	-0.377	0.709	0.709
Complexity	Global within-network mean	Sleep N2	0.283	0.278	-0.559	0.002	-3.010	0.005	0.012
Complexity	Global within-network mean	PPF1.9	0.288	0.277	-1.068	0.003	-3.995	0.002	0.005
Complexity	Global within-network mean	PPF2.4	0.181	0.168	-1.739	0.001	-8.869	0.000	0.000
Complexity	Global within-network mean	PPF2.7	0.234	0.223	-1.216	0.002	-6.321	0.000	0.000

Abbreviations: FC: functional connectivity; LSD: lysergic acid diethylamide; PSIL: psilocybin; KTM: ketamine; N_2O: nitrous oxide; PPF1.9 / PPF2.4 / PPF2.7: propofol (effect-site concentrations: 1.9 / 2.4 / 2.7 μg·mL^{-1}).

bioRxiv preprint doi: https://doi.org/10.64898/2026.02.17.706398; this version posted February 18, 2026. The copyright holder for this preprint (which was not certified by peer review) is the author/funder, who has granted bioRxiv a license to display the preprint in perpetuity. It is made available under aCC-BY 4.0 International license.

Supplementary Table S4. Hierarchical results along the unimodal–attention–transmodal (UAT) axis

Metric	Pair	State	Baseline mean	State mean	Cohen's d	SE	t	p_unc	p_FDR
FC	U-U	LSD	0.325	0.265	-0.821	0.019	-3.179	0.007	0.015
FC	U-U	PSIL	0.586	0.675	0.442	0.076	1.169	0.287	0.368
FC	U-U	KTM	0.338	0.344	0.126	0.013	0.438	0.670	0.754
FC	U-U	N2O	0.443	0.440	-0.016	0.038	-0.060	0.953	0.953
FC	U-U	Sleep N1	0.545	0.524	-0.221	0.016	-1.271	0.213	0.319
FC	U-U	Sleep N2	0.546	0.472	-0.687	0.020	-3.702	0.001	0.003
FC	U-U	PPF1.9	0.519	0.396	-0.651	0.051	-2.437	0.030	0.054
FC	U-U	PPF2.4	0.482	0.340	-1.266	0.022	-6.454	0.000	0.000
FC	U-U	PPF2.7	0.592	0.294	-1.987	0.029	-10.325	0.000	0.000
FC	A-A	LSD	0.278	0.292	0.145	0.024	0.561	0.583	0.601
FC	A-A	PSIL	0.619	0.721	0.538	0.071	1.423	0.205	0.368
FC	A-A	KTM	0.343	0.354	0.214	0.015	0.740	0.475	0.601
FC	A-A	N2O	0.476	0.500	0.138	0.045	0.535	0.601	0.601
FC	A-A	Sleep N1	0.489	0.469	-0.261	0.014	-1.500	0.143	0.323
FC	A-A	Sleep N2	0.491	0.423	-0.713	0.018	-3.839	0.001	0.003
FC	A-A	PPF1.9	0.453	0.409	-0.235	0.051	-0.880	0.395	0.592
FC	A-A	PPF2.4	0.382	0.338	-0.489	0.018	-2.496	0.020	0.059
FC	A-A	PPF2.7	0.513	0.264	-1.473	0.033	-7.654	0.000	0.000
FC	T-T	LSD	0.148	0.187	1.089	0.009	4.219	0.001	0.003
FC	T-T	PSIL	0.572	0.668	0.573	0.063	1.517	0.180	0.232
FC	T-T	KTM	0.268	0.291	0.575	0.012	1.992	0.072	0.161
FC	T-T	N2O	0.377	0.432	0.450	0.032	1.744	0.103	0.186
FC	T-T	Sleep N1	0.456	0.439	-0.247	0.012	-1.419	0.166	0.232
FC	T-T	Sleep N2	0.458	0.399	-0.763	0.014	-4.108	0.000	0.001
FC	T-T	PPF1.9	0.407	0.398	-0.063	0.037	-0.237	0.816	0.816
FC	T-T	PPF2.4	0.321	0.310	-0.143	0.015	-0.728	0.474	0.533
FC	T-T	PPF2.7	0.432	0.282	-0.988	0.029	-5.132	0.000	0.000
FC	U-A	LSD	0.201	0.213	0.159	0.020	0.616	0.548	0.548
FC	U-A	PSIL	0.558	0.677	0.577	0.078	1.527	0.178	0.272
FC	U-A	KTM	0.273	0.299	0.545	0.014	1.890	0.085	0.192
FC	U-A	N2O	0.407	0.437	0.177	0.044	0.686	0.504	0.548
FC	U-A	Sleep N1	0.465	0.445	-0.208	0.016	-1.198	0.240	0.308
FC	U-A	Sleep N2	0.466	0.391	-0.676	0.020	-3.643	0.001	0.003
FC	U-A	PPF1.9	0.418	0.341	-0.377	0.055	-1.412	0.181	0.272
FC	U-A	PPF2.4	0.345	0.266	-0.762	0.020	-3.886	0.001	0.003
FC	U-A	PPF2.7	0.482	0.205	-1.760	0.030	-9.148	0.000	0.000
FC	U-T	LSD	-0.018	0.022	0.842	0.012	3.260	0.006	0.014
FC	U-T	PSIL	0.508	0.625	0.507	0.087	1.342	0.228	0.293
FC	U-T	KTM	0.142	0.206	1.071	0.017	3.710	0.003	0.014
FC	U-T	N2O	0.307	0.369	0.425	0.038	1.645	0.122	0.183
FC	U-T	Sleep N1	0.377	0.371	-0.058	0.019	-0.335	0.740	0.740
FC	U-T	Sleep N2	0.380	0.324	-0.477	0.022	-2.569	0.016	0.028
FC	U-T	PPF1.9	0.309	0.254	-0.258	0.058	-0.964	0.353	0.397
FC	U-T	PPF2.4	0.225	0.163	-0.585	0.021	-2.981	0.006	0.014
FC	U-T	PPF2.7	0.342	0.095	-1.487	0.032	-7.725	0.000	0.000
FC	A-T	LSD	-0.003	0.045	0.990	0.012	3.835	0.002	0.008
FC	A-T	PSIL	0.516	0.643	0.542	0.089	1.433	0.202	0.303
FC	A-T	KTM	0.198	0.244	1.089	0.012	3.774	0.003	0.009
FC	A-T	N2O	0.328	0.396	0.432	0.041	1.675	0.116	0.209
FC	A-T	Sleep N1	0.384	0.375	-0.088	0.018	-0.508	0.615	0.692
FC	A-T	Sleep N2	0.387	0.331	-0.513	0.020	-2.764	0.010	0.022
FC	A-T	PPF1.9	0.328	0.319	-0.041	0.057	-0.152	0.881	0.881
FC	A-T	PPF2.4	0.248	0.235	-0.128	0.019	-0.652	0.520	0.669
FC	A-T	PPF2.7	0.364	0.168	-1.086	0.035	-5.644	0.000	0.000
Efficiency	U-U	LSD	0.687	0.735	0.855	0.015	3.313	0.005	0.009
Efficiency	U-U	PSIL	0.742	0.812	0.752	0.035	1.990	0.094	0.121
Efficiency	U-U	KTM	0.752	0.759	0.219	0.009	0.758	0.464	0.522
Efficiency	U-U	N2O	0.709	0.710	0.056	0.007	0.217	0.831	0.831
Efficiency	U-U	Sleep N1	0.694	0.671	-0.552	0.007	-3.174	0.003	0.007
Efficiency	U-U	Sleep N2	0.695	0.628	-1.237	0.010	-6.659	0.000	0.000
Efficiency	U-U	PPF1.9	0.685	0.606	-1.407	0.015	-5.264	0.000	0.000

bioRxiv preprint doi: https://doi.org/10.64898/2026.02.17.706398; this version posted February 18, 2026. The copyright holder for this preprint (which was not certified by peer review) is the author/funder, who has granted bioRxiv a license to display the preprint in perpetuity. It is made available under aCC-BY 4.0 International license.

Efficiency	U-U	PPF2.4	0.703	0.645	-0.870	0.013	-4.435	0.000	0.000
Efficiency	U-U	PPF2.7	0.701	0.637	-0.551	0.022	-2.864	0.008	0.012
Efficiency	A-A	LSD	0.749	0.798	1.006	0.012	3.895	0.002	0.004
Efficiency	A-A	PSIL	0.844	0.887	0.949	0.017	2.511	0.046	0.059
Efficiency	A-A	KTM	0.788	0.819	0.625	0.014	2.164	0.053	0.060
Efficiency	A-A	N2O	0.822	0.813	-0.234	0.010	-0.906	0.380	0.380
Efficiency	A-A	Sleep N1	0.803	0.788	-0.527	0.005	-3.027	0.005	0.007
Efficiency	A-A	Sleep N2	0.801	0.769	-0.753	0.008	-4.057	0.000	0.001
Efficiency	A-A	PPF1.9	0.779	0.708	-1.421	0.013	-5.318	0.000	0.001
Efficiency	A-A	PPF2.4	0.797	0.764	-0.650	0.010	-3.314	0.003	0.005
Efficiency	A-A	PPF2.7	0.815	0.760	-0.813	0.013	-4.225	0.000	0.001
Efficiency	T-T	LSD	0.746	0.789	1.162	0.009	4.500	0.000	0.003
Efficiency	T-T	PSIL	0.823	0.849	0.350	0.028	0.927	0.390	0.491
Efficiency	T-T	KTM	0.780	0.795	0.381	0.011	1.321	0.213	0.384
Efficiency	T-T	N2O	0.777	0.769	-0.207	0.009	-0.800	0.437	0.491
Efficiency	T-T	Sleep N1	0.793	0.790	-0.099	0.006	-0.571	0.572	0.572
Efficiency	T-T	Sleep N2	0.790	0.772	-0.422	0.008	-2.274	0.031	0.069
Efficiency	T-T	PPF1.9	0.769	0.709	-1.092	0.015	-4.084	0.001	0.004
Efficiency	T-T	PPF2.4	0.783	0.776	-0.203	0.007	-1.033	0.312	0.467
Efficiency	T-T	PPF2.7	0.774	0.728	-0.743	0.012	-3.861	0.001	0.003
Efficiency	U-A	LSD	0.648	0.719	1.319	0.014	5.109	0.000	0.001
Efficiency	U-A	PSIL	0.740	0.806	0.941	0.027	2.491	0.047	0.071
Efficiency	U-A	KTM	0.700	0.711	0.273	0.011	0.947	0.364	0.409
Efficiency	U-A	N2O	0.694	0.692	-0.054	0.009	-0.210	0.836	0.836
Efficiency	U-A	Sleep N1	0.660	0.645	-0.462	0.006	-2.651	0.012	0.027
Efficiency	U-A	Sleep N2	0.661	0.610	-1.104	0.009	-5.947	0.000	0.000
Efficiency	U-A	PPF1.9	0.626	0.572	-1.361	0.011	-5.093	0.000	0.001
Efficiency	U-A	PPF2.4	0.658	0.625	-0.513	0.013	-2.617	0.015	0.027
Efficiency	U-A	PPF2.7	0.660	0.623	-0.379	0.019	-1.967	0.060	0.077
Efficiency	U-T	LSD	0.591	0.667	1.268	0.016	4.909	0.000	0.001
Efficiency	U-T	PSIL	0.734	0.789	0.586	0.036	1.549	0.172	0.222
Efficiency	U-T	KTM	0.683	0.703	0.604	0.009	2.091	0.061	0.091
Efficiency	U-T	N2O	0.687	0.703	0.528	0.008	2.044	0.060	0.091
Efficiency	U-T	Sleep N1	0.656	0.645	-0.359	0.005	-2.065	0.047	0.091
Efficiency	U-T	Sleep N2	0.657	0.619	-0.802	0.009	-4.321	0.000	0.001
Efficiency	U-T	PPF1.9	0.608	0.580	-0.570	0.013	-2.131	0.053	0.091
Efficiency	U-T	PPF2.4	0.631	0.630	-0.006	0.016	-0.031	0.976	0.976
Efficiency	U-T	PPF2.7	0.616	0.603	-0.129	0.019	-0.672	0.508	0.571
Efficiency	A-T	LSD	0.656	0.699	1.048	0.011	4.060	0.001	0.004
Efficiency	A-T	PSIL	0.774	0.804	0.345	0.033	0.912	0.397	0.510
Efficiency	A-T	KTM	0.707	0.729	0.487	0.013	1.688	0.120	0.179
Efficiency	A-T	N2O	0.731	0.725	-0.162	0.009	-0.628	0.540	0.608
Efficiency	A-T	Sleep N1	0.735	0.734	-0.049	0.004	-0.284	0.778	0.778
Efficiency	A-T	Sleep N2	0.734	0.720	-0.424	0.006	-2.285	0.030	0.068
Efficiency	A-T	PPF1.9	0.686	0.631	-1.154	0.013	-4.318	0.001	0.004
Efficiency	A-T	PPF2.4	0.728	0.709	-0.361	0.010	-1.841	0.077	0.139
Efficiency	A-T	PPF2.7	0.720	0.669	-0.737	0.013	-3.831	0.001	0.004
Complexity	U-U	LSD	0.203	0.204	0.149	0.002	0.577	0.573	0.573
Complexity	U-U	PSIL	0.211	0.216	0.565	0.003	1.496	0.185	0.322
Complexity	U-U	KTM	0.247	0.243	-0.380	0.002	-1.318	0.214	0.322
Complexity	U-U	N2O	0.230	0.227	-0.284	0.003	-1.099	0.290	0.330
Complexity	U-U	Sleep N1	0.228	0.227	-0.186	0.001	-1.069	0.293	0.330
Complexity	U-U	Sleep N2	0.228	0.223	-0.521	0.002	-2.808	0.009	0.020
Complexity	U-U	PPF1.9	0.234	0.224	-1.371	0.002	-5.129	0.000	0.001
Complexity	U-U	PPF2.4	0.147	0.131	-1.760	0.002	-8.974	0.000	0.000
Complexity	U-U	PPF2.7	0.191	0.178	-1.275	0.002	-6.624	0.000	0.000
Complexity	A-A	LSD	0.216	0.220	0.360	0.003	1.393	0.185	0.334
Complexity	A-A	PSIL	0.228	0.232	0.351	0.004	0.930	0.388	0.466
Complexity	A-A	KTM	0.264	0.266	0.245	0.002	0.849	0.414	0.466
Complexity	A-A	N2O	0.251	0.251	-0.057	0.004	-0.220	0.829	0.829
Complexity	A-A	Sleep N1	0.250	0.249	-0.159	0.001	-0.911	0.369	0.466
Complexity	A-A	Sleep N2	0.250	0.244	-0.611	0.002	-3.288	0.003	0.006
Complexity	A-A	PPF1.9	0.255	0.242	-1.241	0.003	-4.644	0.000	0.001
Complexity	A-A	PPF2.4	0.154	0.141	-1.478	0.002	-7.536	0.000	0.000
Complexity	A-A	PPF2.7	0.204	0.189	-1.514	0.002	-7.867	0.000	0.000
Complexity	T-T	LSD	0.184	0.193	1.151	0.002	4.459	0.001	0.002
Complexity	T-T	PSIL	0.207	0.212	0.437	0.005	1.156	0.292	0.375

bioRxiv preprint doi: https://doi.org/10.64898/2026.02.17.706398; this version posted February 18, 2026. The copyright holder for this preprint (which was not certified by peer review) is the author/funder, who has granted bioRxiv a license to display the preprint in perpetuity. It is made available under aCC-BY 4.0 International license.

Complexity	T-T	KTM	0.235	0.239	0.406	0.002	1.408	0.187	0.280
Complexity	T-T	N2O	0.229	0.227	-0.102	0.003	-0.396	0.698	0.785
Complexity	T-T	Sleep N1	0.226	0.226	-0.017	0.001	-0.095	0.925	0.925
Complexity	T-T	Sleep N2	0.226	0.220	-0.668	0.002	-3.599	0.001	0.003
Complexity	T-T	PPF1.9	0.228	0.220	-0.829	0.003	-3.101	0.008	0.015
Complexity	T-T	PPF2.4	0.136	0.127	-1.019	0.002	-5.196	0.000	0.000
Complexity	T-T	PPF2.7	0.182	0.173	-1.177	0.001	-6.117	0.000	0.000
Complexity	U-A	LSD	0.207	0.213	0.477	0.003	1.846	0.086	0.155
Complexity	U-A	PSIL	0.223	0.231	0.598	0.005	1.583	0.164	0.247
Complexity	U-A	KTM	0.258	0.260	0.184	0.003	0.636	0.538	0.616
Complexity	U-A	N2O	0.246	0.245	-0.090	0.004	-0.347	0.734	0.734
Complexity	U-A	Sleep N1	0.243	0.242	-0.106	0.001	-0.607	0.548	0.616
Complexity	U-A	Sleep N2	0.243	0.236	-0.673	0.002	-3.623	0.001	0.003
Complexity	U-A	PPF1.9	0.247	0.234	-1.106	0.003	-4.140	0.001	0.003
Complexity	U-A	PPF2.4	0.146	0.132	-1.276	0.002	-6.506	0.000	0.000
Complexity	U-A	PPF2.7	0.197	0.181	-1.393	0.002	-7.239	0.000	0.000
Complexity	U-T	LSD	0.179	0.187	1.082	0.002	4.191	0.001	0.002
Complexity	U-T	PSIL	0.203	0.211	0.543	0.005	1.435	0.201	0.302
Complexity	U-T	KTM	0.229	0.232	0.302	0.003	1.048	0.317	0.408
Complexity	U-T	N2O	0.222	0.222	0.011	0.003	0.041	0.968	0.968
Complexity	U-T	Sleep N1	0.218	0.218	-0.017	0.001	-0.098	0.923	0.968
Complexity	U-T	Sleep N2	0.218	0.213	-0.694	0.002	-3.739	0.001	0.002
Complexity	U-T	PPF1.9	0.220	0.211	-0.809	0.003	-3.026	0.010	0.018
Complexity	U-T	PPF2.4	0.128	0.118	-1.015	0.002	-5.176	0.000	0.000
Complexity	U-T	PPF2.7	0.172	0.163	-1.081	0.002	-5.617	0.000	0.000
Complexity	A-T	LSD	0.191	0.199	1.026	0.002	3.973	0.001	0.004
Complexity	A-T	PSIL	0.219	0.226	0.390	0.007	1.031	0.342	0.440
Complexity	A-T	KTM	0.250	0.254	0.546	0.002	1.891	0.085	0.128
Complexity	A-T	N2O	0.239	0.241	0.117	0.004	0.452	0.658	0.658
Complexity	A-T	Sleep N1	0.239	0.240	0.144	0.001	0.826	0.415	0.467
Complexity	A-T	Sleep N2	0.239	0.234	-0.540	0.002	-2.909	0.007	0.016
Complexity	A-T	PPF1.9	0.242	0.232	-0.731	0.004	-2.737	0.017	0.031
Complexity	A-T	PPF2.4	0.139	0.130	-0.925	0.002	-4.716	0.000	0.000
Complexity	A-T	PPF2.7	0.189	0.177	-1.168	0.002	-6.068	0.000	0.000

Abbreviations: FC: functional connectivity; LSD: lysergic acid diethylamide; PSIL: psilocybin; KTM: ketamine; N_2O: nitrous oxide; PPF1.9 / PPF2.4 / PPF2.7: propofol (effect-site concentrations: 1.9 / 2.4 / 2.7 μg·mL^{-1}). U-U: unimodal-unimodal; A-A: attention-attention; T-T: transmodal-transmodal; U-A: unimodal-attention; U-T: unimodal-transmodal; A-T: attention-transmodal.

bioRxiv preprint doi: https://doi.org/10.64898/2026.02.17.706398; this version posted February 18, 2026. The copyright holder for this preprint (which was not certified by peer review) is the author/funder, who has granted bioRxiv a license to display the preprint in perpetuity. It is made available under aCC-BY 4.0 International license.

Supplementary Table S5. Within- and between-network results at the 7-network level

Metric	Net1	Net2	Edge Type	State	Baseline mean	State mean	Cohen's d	SE	t	p_unc	p_FDR
FC	VIS	VIS	within	LSD	0.315	0.285	-0.465	0.017	-1.800	0.094	0.168
FC	VIS	VIS	within	PSIL	0.655	0.703	0.252	0.073	0.665	0.530	0.682
FC	VIS	VIS	within	KTM	0.410	0.405	-0.072	0.017	-0.248	0.808	0.907
FC	VIS	VIS	within	N2O	0.523	0.527	0.031	0.027	0.119	0.907	0.907
FC	VIS	VIS	within	Sleep N1	0.635	0.615	-0.223	0.016	-1.282	0.209	0.314
FC	VIS	VIS	within	Sleep N2	0.638	0.549	-0.822	0.020	-4.426	0.000	0.000
FC	VIS	VIS	within	PPF1.9	0.567	0.459	-0.676	0.043	-2.529	0.025	0.057
FC	VIS	VIS	within	PPF2.4	0.534	0.422	-1.174	0.019	-5.986	0.000	0.000
FC	VIS	VIS	within	PPF2.7	0.648	0.402	-1.766	0.027	-9.174	0.000	0.000
FC	VIS	SMN	between	LSD	0.220	0.174	-0.546	0.022	-2.114	0.053	0.095
FC	VIS	SMN	between	PSIL	0.524	0.632	0.455	0.090	1.203	0.274	0.316
FC	VIS	SMN	between	KTM	0.254	0.271	0.328	0.015	1.135	0.281	0.316
FC	VIS	SMN	between	N2O	0.349	0.352	0.018	0.043	0.069	0.946	0.946
FC	VIS	SMN	between	Sleep N1	0.460	0.437	-0.213	0.019	-1.222	0.231	0.316
FC	VIS	SMN	between	Sleep N2	0.463	0.376	-0.670	0.024	-3.610	0.001	0.004
FC	VIS	SMN	between	PPF1.9	0.448	0.294	-0.726	0.057	-2.717	0.018	0.040
FC	VIS	SMN	between	PPF2.4	0.413	0.239	-1.401	0.024	-7.145	0.000	0.000
FC	VIS	SMN	between	PPF2.7	0.523	0.192	-2.158	0.030	-11.212	0.000	0.000
FC	VIS	DAN	between	LSD	0.141	0.175	0.530	0.017	2.052	0.059	0.107
FC	VIS	DAN	between	PSIL	0.570	0.669	0.470	0.080	1.243	0.260	0.335
FC	VIS	DAN	between	KTM	0.235	0.273	0.818	0.013	2.833	0.016	0.037
FC	VIS	DAN	between	N2O	0.378	0.412	0.204	0.043	0.790	0.443	0.443
FC	VIS	DAN	between	Sleep N1	0.478	0.460	-0.179	0.017	-1.031	0.310	0.349
FC	VIS	DAN	between	Sleep N2	0.482	0.381	-0.764	0.025	-4.114	0.000	0.001
FC	VIS	DAN	between	PPF1.9	0.416	0.338	-0.399	0.053	-1.494	0.159	0.239
FC	VIS	DAN	between	PPF2.4	0.369	0.275	-0.821	0.022	-4.189	0.000	0.001
FC	VIS	DAN	between	PPF2.7	0.488	0.243	-1.650	0.029	-8.575	0.000	0.000
FC	VIS	VAN	between	LSD	0.118	0.148	0.328	0.023	1.270	0.225	0.325
FC	VIS	VAN	between	PSIL	0.521	0.631	0.441	0.094	1.166	0.288	0.325
FC	VIS	VAN	between	KTM	0.190	0.228	0.679	0.016	2.352	0.038	0.086
FC	VIS	VAN	between	N2O	0.337	0.347	0.061	0.040	0.238	0.815	0.815
FC	VIS	VAN	between	Sleep N1	0.380	0.361	-0.188	0.017	-1.079	0.289	0.325
FC	VIS	VAN	between	Sleep N2	0.378	0.317	-0.549	0.021	-2.958	0.006	0.019
FC	VIS	VAN	between	PPF1.9	0.327	0.247	-0.412	0.052	-1.541	0.147	0.265
FC	VIS	VAN	between	PPF2.4	0.263	0.191	-0.722	0.020	-3.680	0.001	0.005
FC	VIS	VAN	between	PPF2.7	0.405	0.146	-1.657	0.030	-8.609	0.000	0.000
FC	VIS	LIM	between	LSD	0.036	0.032	-0.072	0.011	-0.281	0.783	0.848
FC	VIS	LIM	between	PSIL	0.462	0.531	0.293	0.090	0.775	0.468	0.601
FC	VIS	LIM	between	KTM	0.154	0.192	0.628	0.018	2.176	0.052	0.094
FC	VIS	LIM	between	N2O	0.259	0.233	-0.205	0.033	-0.793	0.441	0.601
FC	VIS	LIM	between	Sleep N1	0.346	0.342	-0.034	0.020	-0.193	0.848	0.848
FC	VIS	LIM	between	Sleep N2	0.344	0.298	-0.479	0.018	-2.581	0.015	0.035
FC	VIS	LIM	between	PPF1.9	0.311	0.165	-0.915	0.042	-3.425	0.005	0.014
FC	VIS	LIM	between	PPF2.4	0.305	0.182	-1.410	0.017	-7.190	0.000	0.000
FC	VIS	LIM	between	PPF2.7	0.300	0.089	-2.105	0.019	-10.938	0.000	0.000
FC	VIS	FPN	between	LSD	-0.025	0.042	0.973	0.018	3.769	0.002	0.006
FC	VIS	FPN	between	PSIL	0.514	0.629	0.532	0.081	1.408	0.209	0.269
FC	VIS	FPN	between	KTM	0.114	0.184	0.925	0.022	3.206	0.008	0.019
FC	VIS	FPN	between	N2O	0.282	0.349	0.543	0.032	2.102	0.054	0.089
FC	VIS	FPN	between	Sleep N1	0.350	0.337	-0.140	0.017	-0.805	0.427	0.480
FC	VIS	FPN	between	Sleep N2	0.351	0.284	-0.674	0.018	-3.627	0.001	0.005
FC	VIS	FPN	between	PPF1.9	0.279	0.246	-0.193	0.045	-0.724	0.482	0.482
FC	VIS	FPN	between	PPF2.4	0.214	0.179	-0.388	0.017	-1.978	0.059	0.089
FC	VIS	FPN	between	PPF2.7	0.318	0.133	-1.110	0.032	-5.768	0.000	0.000
FC	VIS	DMN	between	LSD	0.016	0.056	0.979	0.011	3.790	0.002	0.005
FC	VIS	DMN	between	PSIL	0.504	0.615	0.541	0.077	1.431	0.202	0.260
FC	VIS	DMN	between	KTM	0.140	0.205	1.008	0.019	3.493	0.005	0.009
FC	VIS	DMN	between	N2O	0.322	0.356	0.269	0.033	1.041	0.316	0.355
FC	VIS	DMN	between	Sleep N1	0.402	0.391	-0.099	0.019	-0.569	0.573	0.573
FC	VIS	DMN	between	Sleep N2	0.403	0.328	-0.632	0.022	-3.406	0.002	0.005
FC	VIS	DMN	between	PPF1.9	0.314	0.228	-0.416	0.055	-1.555	0.144	0.216
FC	VIS	DMN	between	PPF2.4	0.245	0.165	-0.817	0.019	-4.165	0.000	0.001

bioRxiv preprint doi: https://doi.org/10.64898/2026.02.17.706398; this version posted February 18, 2026. The copyright holder for this preprint (which was not certified by peer review) is the author/funder, who has granted bioRxiv a license to display the preprint in perpetuity. It is made available under aCC-BY 4.0 International license.

FC	VIS	DMN	between	PPF2.7	0.355	0.105	-1.553	0.031	-8.068	0.000	0.000
FC	SMN	SMN	within	LSD	0.497	0.397	-0.909	0.028	-3.521	0.003	0.010
FC	SMN	SMN	within	PSIL	0.641	0.724	0.506	0.062	1.338	0.229	0.344
FC	SMN	SMN	within	KTM	0.427	0.420	-0.125	0.015	-0.433	0.674	0.758
FC	SMN	SMN	within	N2O	0.540	0.526	-0.079	0.046	-0.306	0.764	0.764
FC	SMN	SMN	within	Sleep N1	0.621	0.605	-0.196	0.014	-1.125	0.269	0.346
FC	SMN	SMN	within	Sleep N2	0.621	0.576	-0.463	0.018	-2.495	0.019	0.042
FC	SMN	SMN	within	PPF1.9	0.603	0.519	-0.421	0.053	-1.574	0.139	0.251
FC	SMN	SMN	within	PPF2.4	0.557	0.450	-0.849	0.025	-4.331	0.000	0.001
FC	SMN	SMN	within	PPF2.7	0.666	0.386	-1.623	0.033	-8.432	0.000	0.000
FC	SMN	DAN	between	LSD	0.220	0.232	0.101	0.033	0.391	0.702	0.702
FC	SMN	DAN	between	PSIL	0.560	0.691	0.658	0.076	1.741	0.132	0.206
FC	SMN	DAN	between	KTM	0.308	0.335	0.489	0.016	1.693	0.119	0.206
FC	SMN	DAN	between	N2O	0.433	0.487	0.295	0.047	1.141	0.273	0.307
FC	SMN	DAN	between	Sleep N1	0.503	0.478	-0.250	0.017	-1.437	0.161	0.206
FC	SMN	DAN	between	Sleep N2	0.507	0.408	-0.758	0.024	-4.080	0.000	0.002
FC	SMN	DAN	between	PPF1.9	0.462	0.363	-0.419	0.064	-1.567	0.141	0.206
FC	SMN	DAN	between	PPF2.4	0.368	0.274	-0.765	0.024	-3.899	0.001	0.002
FC	SMN	DAN	between	PPF2.7	0.503	0.182	-2.053	0.030	-10.666	0.000	0.000
FC	SMN	VAN	between	LSD	0.294	0.275	-0.207	0.024	-0.800	0.437	0.562
FC	SMN	VAN	between	PSIL	0.576	0.705	0.653	0.075	1.727	0.135	0.303
FC	SMN	VAN	between	KTM	0.333	0.338	0.094	0.017	0.324	0.752	0.752
FC	SMN	VAN	between	N2O	0.458	0.478	0.103	0.050	0.398	0.697	0.752
FC	SMN	VAN	between	Sleep N1	0.484	0.469	-0.169	0.016	-0.969	0.340	0.562
FC	SMN	VAN	between	Sleep N2	0.482	0.443	-0.392	0.019	-2.110	0.044	0.132
FC	SMN	VAN	between	PPF1.9	0.449	0.396	-0.241	0.059	-0.902	0.383	0.562
FC	SMN	VAN	between	PPF2.4	0.370	0.311	-0.505	0.023	-2.576	0.016	0.073
FC	SMN	VAN	between	PPF2.7	0.517	0.245	-1.456	0.036	-7.564	0.000	0.000
FC	SMN	LIM	between	LSD	0.024	0.015	-0.137	0.018	-0.531	0.604	0.827
FC	SMN	LIM	between	PSIL	0.479	0.528	0.184	0.100	0.488	0.643	0.827
FC	SMN	LIM	between	KTM	0.160	0.179	0.231	0.024	0.802	0.440	0.791
FC	SMN	LIM	between	N2O	0.183	0.182	-0.008	0.042	-0.031	0.976	0.976
FC	SMN	LIM	between	Sleep N1	0.320	0.319	-0.007	0.019	-0.039	0.969	0.976
FC	SMN	LIM	between	Sleep N2	0.319	0.290	-0.278	0.019	-1.497	0.146	0.328
FC	SMN	LIM	between	PPF1.9	0.319	0.144	-0.842	0.055	-3.152	0.008	0.023
FC	SMN	LIM	between	PPF2.4	0.294	0.191	-0.900	0.023	-4.590	0.000	0.000
FC	SMN	LIM	between	PPF2.7	0.292	0.075	-2.247	0.019	-11.674	0.000	0.000
FC	SMN	FPN	between	LSD	-0.040	0.014	0.520	0.027	2.013	0.064	0.144
FC	SMN	FPN	between	PSIL	0.520	0.639	0.504	0.089	1.332	0.231	0.297
FC	SMN	FPN	between	KTM	0.161	0.220	0.839	0.020	2.906	0.014	0.053
FC	SMN	FPN	between	N2O	0.310	0.391	0.474	0.044	1.837	0.088	0.158
FC	SMN	FPN	between	Sleep N1	0.361	0.352	-0.079	0.019	-0.454	0.653	0.653
FC	SMN	FPN	between	Sleep N2	0.367	0.307	-0.468	0.024	-2.522	0.018	0.053
FC	SMN	FPN	between	PPF1.9	0.299	0.269	-0.124	0.064	-0.464	0.651	0.653
FC	SMN	FPN	between	PPF2.4	0.195	0.157	-0.323	0.023	-1.648	0.112	0.168
FC	SMN	FPN	between	PPF2.7	0.336	0.069	-1.421	0.036	-7.384	0.000	0.000
FC	SMN	DMN	between	LSD	-0.029	-0.009	0.324	0.016	1.254	0.230	0.339
FC	SMN	DMN	between	PSIL	0.500	0.623	0.466	0.099	1.234	0.263	0.339
FC	SMN	DMN	between	KTM	0.145	0.209	1.019	0.018	3.528	0.005	0.021
FC	SMN	DMN	between	N2O	0.305	0.376	0.385	0.048	1.492	0.158	0.334
FC	SMN	DMN	between	Sleep N1	0.379	0.381	0.016	0.021	0.091	0.928	0.928
FC	SMN	DMN	between	Sleep N2	0.384	0.348	-0.252	0.026	-1.357	0.186	0.334
FC	SMN	DMN	between	PPF1.9	0.326	0.268	-0.232	0.066	-0.870	0.400	0.450
FC	SMN	DMN	between	PPF2.4	0.231	0.158	-0.554	0.026	-2.824	0.009	0.028
FC	SMN	DMN	between	PPF2.7	0.346	0.085	-1.515	0.033	-7.871	0.000	0.000
FC	DAN	DAN	within	LSD	0.345	0.358	0.163	0.020	0.630	0.539	0.539
FC	DAN	DAN	within	PSIL	0.666	0.747	0.531	0.057	1.405	0.210	0.454
FC	DAN	DAN	within	KTM	0.396	0.413	0.312	0.015	1.081	0.303	0.454
FC	DAN	DAN	within	N2O	0.530	0.562	0.202	0.042	0.781	0.448	0.504
FC	DAN	DAN	within	Sleep N1	0.573	0.559	-0.187	0.013	-1.076	0.290	0.454
FC	DAN	DAN	within	Sleep N2	0.580	0.498	-0.795	0.019	-4.280	0.000	0.001
FC	DAN	DAN	within	PPF1.9	0.544	0.500	-0.242	0.049	-0.904	0.383	0.492
FC	DAN	DAN	within	PPF2.4	0.464	0.425	-0.436	0.018	-2.221	0.036	0.107
FC	DAN	DAN	within	PPF2.7	0.579	0.373	-1.416	0.028	-7.360	0.000	0.000
FC	DAN	VAN	between	LSD	0.191	0.216	0.205	0.032	0.793	0.441	0.459
FC	DAN	VAN	between	PSIL	0.582	0.698	0.552	0.079	1.462	0.194	0.349
FC	DAN	VAN	between	KTM	0.292	0.313	0.328	0.018	1.135	0.281	0.421

bioRxiv preprint doi: https://doi.org/10.64898/2026.02.17.706398; this version posted February 18, 2026. The copyright holder for this preprint (which was not certified by peer review) is the author/funder, who has granted bioRxiv a license to display the preprint in perpetuity. It is made available under aCC-BY 4.0 International license.

FC	DAN	VAN	between	N2O	0.432	0.468	0.200	0.047	0.774	0.452	0.459
FC	DAN	VAN	between	Sleep N1	0.441	0.417	-0.282	0.015	-1.618	0.116	0.260
FC	DAN	VAN	between	Sleep N2	0.442	0.365	-0.728	0.020	-3.918	0.001	0.002
FC	DAN	VAN	between	PPF1.9	0.395	0.352	-0.204	0.056	-0.763	0.459	0.459
FC	DAN	VAN	between	PPF2.4	0.318	0.278	-0.425	0.019	-2.169	0.040	0.119
FC	DAN	VAN	between	PPF2.7	0.459	0.194	-1.471	0.035	-7.643	0.000	0.000
FC	DAN	LIM	between	LSD	0.003	0.009	0.132	0.013	0.511	0.617	0.794
FC	DAN	LIM	between	PSIL	0.473	0.544	0.273	0.099	0.723	0.497	0.746
FC	DAN	LIM	between	KTM	0.172	0.185	0.209	0.018	0.725	0.483	0.746
FC	DAN	LIM	between	N2O	0.230	0.224	-0.031	0.044	-0.121	0.905	0.905
FC	DAN	LIM	between	Sleep N1	0.342	0.339	-0.033	0.017	-0.191	0.849	0.905
FC	DAN	LIM	between	Sleep N2	0.341	0.297	-0.493	0.017	-2.654	0.013	0.032
FC	DAN	LIM	between	PPF1.9	0.322	0.165	-0.755	0.056	-2.823	0.014	0.032
FC	DAN	LIM	between	PPF2.4	0.282	0.195	-0.813	0.021	-4.147	0.000	0.002
FC	DAN	LIM	between	PPF2.7	0.293	0.100	-1.587	0.023	-8.247	0.000	0.000
FC	DAN	FPN	between	LSD	0.092	0.120	0.399	0.018	1.547	0.144	0.260
FC	DAN	FPN	between	PSIL	0.583	0.679	0.507	0.072	1.342	0.228	0.342
FC	DAN	FPN	between	KTM	0.253	0.281	0.493	0.016	1.706	0.116	0.260
FC	DAN	FPN	between	N2O	0.376	0.437	0.419	0.037	1.623	0.127	0.260
FC	DAN	FPN	between	Sleep N1	0.431	0.419	-0.141	0.015	-0.811	0.424	0.545
FC	DAN	FPN	between	Sleep N2	0.434	0.372	-0.665	0.017	-3.582	0.001	0.006
FC	DAN	FPN	between	PPF1.9	0.372	0.378	0.035	0.048	0.132	0.897	0.954
FC	DAN	FPN	between	PPF2.4	0.292	0.293	0.011	0.014	0.058	0.954	0.954
FC	DAN	FPN	between	PPF2.7	0.413	0.241	-1.022	0.032	-5.308	0.000	0.000
FC	DAN	DMN	between	LSD	-0.064	0.002	1.016	0.017	3.936	0.001	0.007
FC	DAN	DMN	between	PSIL	0.485	0.643	0.648	0.092	1.715	0.137	0.176
FC	DAN	DMN	between	KTM	0.170	0.227	1.147	0.015	3.972	0.002	0.007
FC	DAN	DMN	between	N2O	0.304	0.394	0.526	0.044	2.037	0.061	0.110
FC	DAN	DMN	between	Sleep N1	0.389	0.380	-0.084	0.020	-0.484	0.631	0.631
FC	DAN	DMN	between	Sleep N2	0.393	0.322	-0.543	0.024	-2.922	0.007	0.015
FC	DAN	DMN	between	PPF1.9	0.319	0.276	-0.175	0.067	-0.654	0.524	0.590
FC	DAN	DMN	between	PPF2.4	0.230	0.192	-0.333	0.022	-1.697	0.102	0.153
FC	DAN	DMN	between	PPF2.7	0.353	0.121	-1.193	0.038	-6.200	0.000	0.000
FC	VAN	VAN	within	LSD	0.385	0.376	-0.097	0.023	-0.377	0.712	0.801
FC	VAN	VAN	within	PSIL	0.646	0.739	0.498	0.070	1.317	0.236	0.424
FC	VAN	VAN	within	KTM	0.390	0.379	-0.247	0.013	-0.854	0.411	0.529
FC	VAN	VAN	within	N2O	0.512	0.502	-0.055	0.047	-0.212	0.836	0.836
FC	VAN	VAN	within	Sleep N1	0.504	0.483	-0.248	0.014	-1.425	0.164	0.369
FC	VAN	VAN	within	Sleep N2	0.501	0.463	-0.422	0.017	-2.271	0.031	0.093
FC	VAN	VAN	within	PPF1.9	0.480	0.432	-0.270	0.048	-1.009	0.331	0.497
FC	VAN	VAN	within	PPF2.4	0.430	0.372	-0.551	0.021	-2.812	0.009	0.042
FC	VAN	VAN	within	PPF2.7	0.557	0.296	-1.368	0.037	-7.111	0.000	0.000
FC	VAN	LIM	between	LSD	0.021	0.007	-0.276	0.013	-1.069	0.303	0.546
FC	VAN	LIM	between	PSIL	0.466	0.523	0.212	0.102	0.560	0.596	0.894
FC	VAN	LIM	between	KTM	0.170	0.177	0.083	0.023	0.287	0.780	0.961
FC	VAN	LIM	between	N2O	0.170	0.171	0.002	0.045	0.006	0.995	0.995
FC	VAN	LIM	between	Sleep N1	0.275	0.272	-0.032	0.016	-0.185	0.854	0.961
FC	VAN	LIM	between	Sleep N2	0.274	0.253	-0.256	0.016	-1.380	0.179	0.402
FC	VAN	LIM	between	PPF1.9	0.277	0.172	-0.558	0.050	-2.089	0.057	0.171
FC	VAN	LIM	between	PPF2.4	0.243	0.196	-0.412	0.022	-2.102	0.046	0.171
FC	VAN	LIM	between	PPF2.7	0.270	0.106	-1.416	0.022	-7.357	0.000	0.000
FC	VAN	FPN	between	LSD	0.052	0.098	0.526	0.023	2.038	0.061	0.137
FC	VAN	FPN	between	PSIL	0.556	0.651	0.419	0.085	1.109	0.310	0.419
FC	VAN	FPN	between	KTM	0.231	0.262	0.639	0.014	2.212	0.049	0.137
FC	VAN	FPN	between	N2O	0.359	0.403	0.274	0.041	1.060	0.307	0.419
FC	VAN	FPN	between	Sleep N1	0.380	0.365	-0.174	0.016	-0.998	0.326	0.419
FC	VAN	FPN	between	Sleep N2	0.382	0.319	-0.613	0.019	-3.302	0.003	0.012
FC	VAN	FPN	between	PPF1.9	0.330	0.334	0.021	0.053	0.080	0.937	0.937
FC	VAN	FPN	between	PPF2.4	0.262	0.253	-0.107	0.017	-0.545	0.591	0.664
FC	VAN	FPN	between	PPF2.7	0.378	0.170	-1.079	0.037	-5.605	0.000	0.000
FC	VAN	DMN	between	LSD	-0.028	0.014	0.810	0.013	3.135	0.007	0.022
FC	VAN	DMN	between	PSIL	0.485	0.618	0.501	0.100	1.327	0.233	0.349
FC	VAN	DMN	between	KTM	0.176	0.230	1.211	0.013	4.194	0.002	0.007
FC	VAN	DMN	between	N2O	0.307	0.372	0.391	0.043	1.514	0.152	0.274
FC	VAN	DMN	between	Sleep N1	0.356	0.353	-0.022	0.018	-0.126	0.900	0.913
FC	VAN	DMN	between	Sleep N2	0.357	0.324	-0.296	0.021	-1.595	0.122	0.274
FC	VAN	DMN	between	PPF1.9	0.310	0.320	0.045	0.058	0.169	0.869	0.913

bioRxiv preprint doi: https://doi.org/10.64898/2026.02.17.706398; this version posted February 18, 2026. The copyright holder for this preprint (which was not certified by peer review) is the author/funder, who has granted bioRxiv a license to display the preprint in perpetuity. It is made available under aCC-BY 4.0 International license.

FC	VAN	DMN	between	PPF2.4	0.233	0.236	0.022	0.024	0.111	0.913	0.913
FC	VAN	DMN	between	PPF2.7	0.338	0.171	-0.896	0.036	-4.658	0.000	0.001
FC	LIM	LIM	within	LSD	0.186	0.189	0.048	0.016	0.186	0.855	0.855
FC	LIM	LIM	within	PSIL	0.509	0.527	0.084	0.083	0.222	0.832	0.855
FC	LIM	LIM	within	KTM	0.313	0.282	-0.374	0.024	-1.294	0.222	0.496
FC	LIM	LIM	within	N2O	0.443	0.450	0.091	0.020	0.351	0.731	0.855
FC	LIM	LIM	within	Sleep N1	0.426	0.432	0.069	0.016	0.396	0.695	0.855
FC	LIM	LIM	within	Sleep N2	0.421	0.410	-0.206	0.010	-1.112	0.276	0.496
FC	LIM	LIM	within	PPF1.9	0.367	0.296	-0.441	0.043	-1.649	0.123	0.369
FC	LIM	LIM	within	PPF2.4	0.366	0.317	-0.494	0.019	-2.520	0.018	0.083
FC	LIM	LIM	within	PPF2.7	0.283	0.159	-1.430	0.017	-7.431	0.000	0.000
FC	LIM	FPN	between	LSD	0.024	0.042	0.365	0.013	1.413	0.180	0.323
FC	LIM	FPN	between	PSIL	0.483	0.538	0.236	0.088	0.624	0.555	0.714
FC	LIM	FPN	between	KTM	0.166	0.164	-0.034	0.015	-0.117	0.909	0.909
FC	LIM	FPN	between	N2O	0.195	0.221	0.213	0.031	0.826	0.423	0.634
FC	LIM	FPN	between	Sleep N1	0.308	0.305	-0.036	0.014	-0.208	0.837	0.909
FC	LIM	FPN	between	Sleep N2	0.308	0.275	-0.478	0.013	-2.572	0.016	0.050
FC	LIM	FPN	between	PPF1.9	0.299	0.198	-0.549	0.049	-2.053	0.061	0.137
FC	LIM	FPN	between	PPF2.4	0.234	0.194	-0.503	0.016	-2.565	0.017	0.050
FC	LIM	FPN	between	PPF2.7	0.256	0.122	-1.150	0.023	-5.974	0.000	0.000
FC	LIM	DMN	between	LSD	0.084	0.098	0.281	0.013	1.088	0.295	0.531
FC	LIM	DMN	between	PSIL	0.512	0.547	0.178	0.074	0.471	0.654	0.841
FC	LIM	DMN	between	KTM	0.167	0.179	0.221	0.016	0.764	0.461	0.691
FC	LIM	DMN	between	N2O	0.280	0.277	-0.027	0.028	-0.105	0.918	0.918
FC	LIM	DMN	between	Sleep N1	0.345	0.347	0.026	0.015	0.150	0.881	0.918
FC	LIM	DMN	between	Sleep N2	0.344	0.315	-0.441	0.012	-2.374	0.025	0.056
FC	LIM	DMN	between	PPF1.9	0.338	0.213	-0.701	0.048	-2.625	0.021	0.056
FC	LIM	DMN	between	PPF2.4	0.290	0.210	-0.907	0.017	-4.623	0.000	0.000
FC	LIM	DMN	between	PPF2.7	0.293	0.141	-1.395	0.021	-7.250	0.000	0.000
FC	FPN	FPN	within	LSD	0.269	0.262	-0.151	0.013	-0.585	0.568	0.798
FC	FPN	FPN	within	PSIL	0.610	0.692	0.479	0.065	1.267	0.252	0.567
FC	FPN	FPN	within	KTM	0.323	0.319	-0.091	0.012	-0.314	0.759	0.854
FC	FPN	FPN	within	N2O	0.406	0.438	0.267	0.031	1.034	0.319	0.573
FC	FPN	FPN	within	Sleep N1	0.473	0.459	-0.205	0.012	-1.177	0.248	0.567
FC	FPN	FPN	within	Sleep N2	0.473	0.422	-0.659	0.015	-3.547	0.001	0.006
FC	FPN	FPN	within	PPF1.9	0.431	0.448	0.136	0.033	0.507	0.621	0.798
FC	FPN	FPN	within	PPF2.4	0.361	0.361	0.003	0.013	0.017	0.987	0.987
FC	FPN	FPN	within	PPF2.7	0.454	0.324	-0.786	0.032	-4.085	0.000	0.003
FC	FPN	DMN	between	LSD	0.062	0.123	1.521	0.010	5.890	0.000	0.000
FC	FPN	DMN	between	PSIL	0.526	0.647	0.612	0.075	1.619	0.157	0.225
FC	FPN	DMN	between	KTM	0.229	0.266	0.813	0.013	2.815	0.017	0.038
FC	FPN	DMN	between	N2O	0.326	0.403	0.587	0.034	2.272	0.039	0.071
FC	FPN	DMN	between	Sleep N1	0.413	0.393	-0.241	0.014	-1.386	0.175	0.225
FC	FPN	DMN	between	Sleep N2	0.415	0.347	-0.757	0.017	-4.077	0.000	0.001
FC	FPN	DMN	between	PPF1.9	0.354	0.359	0.028	0.044	0.103	0.919	0.919
FC	FPN	DMN	between	PPF2.4	0.276	0.273	-0.028	0.016	-0.143	0.888	0.919
FC	FPN	DMN	between	PPF2.7	0.383	0.232	-0.914	0.032	-4.747	0.000	0.000
FC	DMN	DMN	within	LSD	0.208	0.237	0.552	0.014	2.138	0.051	0.152
FC	DMN	DMN	within	PSIL	0.612	0.683	0.531	0.051	1.404	0.210	0.270
FC	DMN	DMN	within	KTM	0.295	0.312	0.399	0.012	1.384	0.194	0.270
FC	DMN	DMN	within	N2O	0.426	0.463	0.309	0.031	1.195	0.252	0.283
FC	DMN	DMN	within	Sleep N1	0.501	0.484	-0.255	0.011	-1.467	0.152	0.270
FC	DMN	DMN	within	Sleep N2	0.502	0.451	-0.718	0.013	-3.865	0.001	0.003
FC	DMN	DMN	within	PPF1.9	0.459	0.426	-0.254	0.034	-0.950	0.359	0.359
FC	DMN	DMN	within	PPF2.4	0.361	0.336	-0.290	0.017	-1.478	0.152	0.270
FC	DMN	DMN	within	PPF2.7	0.481	0.325	-1.050	0.029	-5.453	0.000	0.000
Efficiency	VIS	VIS	within	LSD	0.780	0.828	1.053	0.012	4.080	0.001	0.005
Efficiency	VIS	VIS	within	PSIL	0.813	0.860	0.498	0.036	1.317	0.236	0.303
Efficiency	VIS	VIS	within	KTM	0.795	0.784	-0.273	0.012	-0.946	0.364	0.410
Efficiency	VIS	VIS	within	N2O	0.780	0.774	-0.115	0.014	-0.445	0.663	0.663
Efficiency	VIS	VIS	within	Sleep N1	0.801	0.783	-0.384	0.008	-2.203	0.035	0.052
Efficiency	VIS	VIS	within	Sleep N2	0.799	0.734	-0.913	0.013	-4.916	0.000	0.000
Efficiency	VIS	VIS	within	PPF1.9	0.757	0.711	-0.676	0.018	-2.529	0.025	0.049
Efficiency	VIS	VIS	within	PPF2.4	0.779	0.749	-0.460	0.013	-2.344	0.027	0.049
Efficiency	VIS	VIS	within	PPF2.7	0.786	0.742	-0.469	0.018	-2.436	0.022	0.049
Efficiency	VIS	SMN	between	LSD	0.657	0.701	0.745	0.015	2.883	0.012	0.018
Efficiency	VIS	SMN	between	PSIL	0.727	0.790	0.700	0.034	1.853	0.113	0.146

bioRxiv preprint doi: https://doi.org/10.64898/2026.02.17.706398; this version posted February 18, 2026. The copyright holder for this preprint (which was not certified by peer review) is the author/funder, who has granted bioRxiv a license to display the preprint in perpetuity. It is made available under aCC-BY 4.0 International license.

Efficiency	VIS	SMN	between	KTM	0.720	0.722	0.065	0.008	0.225	0.826	0.910
Efficiency	VIS	SMN	between	N2O	0.684	0.684	0.030	0.008	0.115	0.910	0.910
Efficiency	VIS	SMN	between	Sleep N1	0.667	0.641	-0.605	0.007	-3.477	0.001	0.003
Efficiency	VIS	SMN	between	Sleep N2	0.668	0.597	-1.262	0.010	-6.798	0.000	0.000
Efficiency	VIS	SMN	between	PPF1.9	0.637	0.549	-1.625	0.014	-6.079	0.000	0.000
Efficiency	VIS	SMN	between	PPF2.4	0.680	0.604	-1.075	0.014	-5.483	0.000	0.000
Efficiency	VIS	SMN	between	PPF2.7	0.675	0.596	-0.668	0.023	-3.469	0.002	0.003
Efficiency	VIS	DAN	between	LSD	0.696	0.757	1.772	0.009	6.861	0.000	0.000
Efficiency	VIS	DAN	between	PSIL	0.766	0.832	0.681	0.036	1.802	0.122	0.156
Efficiency	VIS	DAN	between	KTM	0.715	0.716	0.025	0.012	0.087	0.932	0.932
Efficiency	VIS	DAN	between	N2O	0.728	0.727	-0.038	0.008	-0.147	0.886	0.932
Efficiency	VIS	DAN	between	Sleep N1	0.729	0.718	-0.313	0.006	-1.797	0.082	0.123
Efficiency	VIS	DAN	between	Sleep N2	0.727	0.677	-0.882	0.010	-4.752	0.000	0.000
Efficiency	VIS	DAN	between	PPF1.9	0.677	0.642	-0.584	0.016	-2.185	0.048	0.097
Efficiency	VIS	DAN	between	PPF2.4	0.722	0.695	-0.397	0.013	-2.024	0.054	0.097
Efficiency	VIS	DAN	between	PPF2.7	0.717	0.684	-0.393	0.016	-2.042	0.051	0.097
Efficiency	VIS	VAN	between	LSD	0.694	0.760	1.457	0.012	5.642	0.000	0.001
Efficiency	VIS	VAN	between	PSIL	0.756	0.822	0.630	0.040	1.667	0.146	0.330
Efficiency	VIS	VAN	between	KTM	0.709	0.708	-0.014	0.017	-0.049	0.962	0.962
Efficiency	VIS	VAN	between	N2O	0.722	0.716	-0.110	0.016	-0.424	0.678	0.762
Efficiency	VIS	VAN	between	Sleep N1	0.675	0.663	-0.280	0.007	-1.607	0.118	0.330
Efficiency	VIS	VAN	between	Sleep N2	0.670	0.638	-0.550	0.011	-2.960	0.006	0.028
Efficiency	VIS	VAN	between	PPF1.9	0.635	0.614	-0.334	0.017	-1.249	0.234	0.372
Efficiency	VIS	VAN	between	PPF2.4	0.681	0.664	-0.212	0.016	-1.082	0.289	0.372
Efficiency	VIS	VAN	between	PPF2.7	0.667	0.647	-0.214	0.018	-1.111	0.277	0.372
Efficiency	VIS	LIM	between	LSD	0.647	0.725	1.265	0.016	4.900	0.000	0.002
Efficiency	VIS	LIM	between	PSIL	0.693	0.741	0.372	0.049	0.984	0.363	0.653
Efficiency	VIS	LIM	between	KTM	0.672	0.663	-0.205	0.013	-0.711	0.492	0.738
Efficiency	VIS	LIM	between	N2O	0.645	0.641	-0.055	0.018	-0.212	0.835	0.939
Efficiency	VIS	LIM	between	Sleep N1	0.628	0.625	-0.064	0.008	-0.370	0.714	0.918
Efficiency	VIS	LIM	between	Sleep N2	0.624	0.602	-0.425	0.010	-2.288	0.030	0.135
Efficiency	VIS	LIM	between	PPF1.9	0.604	0.581	-0.452	0.014	-1.689	0.115	0.345
Efficiency	VIS	LIM	between	PPF2.4	0.659	0.643	-0.186	0.017	-0.950	0.351	0.653
Efficiency	VIS	LIM	between	PPF2.7	0.577	0.577	0.008	0.017	0.039	0.969	0.969
Efficiency	VIS	FPN	between	LSD	0.643	0.714	2.361	0.008	9.145	0.000	0.000
Efficiency	VIS	FPN	between	PSIL	0.748	0.809	0.537	0.043	1.422	0.205	0.369
Efficiency	VIS	FPN	between	KTM	0.669	0.684	0.263	0.016	0.909	0.383	0.527
Efficiency	VIS	FPN	between	N2O	0.679	0.699	0.428	0.012	1.656	0.120	0.360
Efficiency	VIS	FPN	between	Sleep N1	0.649	0.645	-0.100	0.007	-0.572	0.571	0.571
Efficiency	VIS	FPN	between	Sleep N2	0.645	0.626	-0.320	0.011	-1.725	0.095	0.360
Efficiency	VIS	FPN	between	PPF1.9	0.611	0.622	0.177	0.017	0.663	0.519	0.571
Efficiency	VIS	FPN	between	PPF2.4	0.647	0.661	0.164	0.017	0.838	0.410	0.527
Efficiency	VIS	FPN	between	PPF2.7	0.617	0.645	0.276	0.020	1.434	0.164	0.368
Efficiency	VIS	DMN	between	LSD	0.659	0.719	2.035	0.008	7.882	0.000	0.000
Efficiency	VIS	DMN	between	PSIL	0.746	0.793	0.394	0.044	1.043	0.337	0.506
Efficiency	VIS	DMN	between	KTM	0.677	0.689	0.290	0.013	1.004	0.337	0.506
Efficiency	VIS	DMN	between	N2O	0.701	0.697	-0.102	0.010	-0.395	0.699	0.699
Efficiency	VIS	DMN	between	Sleep N1	0.681	0.676	-0.119	0.007	-0.682	0.500	0.613
Efficiency	VIS	DMN	between	Sleep N2	0.676	0.642	-0.587	0.011	-3.159	0.004	0.017
Efficiency	VIS	DMN	between	PPF1.9	0.628	0.592	-0.809	0.012	-3.026	0.010	0.029
Efficiency	VIS	DMN	between	PPF2.4	0.668	0.658	-0.120	0.017	-0.613	0.545	0.613
Efficiency	VIS	DMN	between	PPF2.7	0.643	0.613	-0.335	0.017	-1.742	0.093	0.210
Efficiency	SMN	SMN	within	LSD	0.781	0.787	0.123	0.014	0.475	0.642	0.723
Efficiency	SMN	SMN	within	PSIL	0.810	0.863	1.175	0.017	3.109	0.021	0.031
Efficiency	SMN	SMN	within	KTM	0.832	0.838	0.219	0.009	0.760	0.463	0.596
Efficiency	SMN	SMN	within	N2O	0.783	0.783	0.006	0.009	0.022	0.983	0.983
Efficiency	SMN	SMN	within	Sleep N1	0.770	0.750	-0.700	0.005	-4.020	0.000	0.001
Efficiency	SMN	SMN	within	Sleep N2	0.770	0.716	-0.899	0.011	-4.840	0.000	0.000
Efficiency	SMN	SMN	within	PPF1.9	0.760	0.682	-1.525	0.014	-5.704	0.000	0.000
Efficiency	SMN	SMN	within	PPF2.4	0.755	0.717	-0.687	0.011	-3.505	0.002	0.004
Efficiency	SMN	SMN	within	PPF2.7	0.767	0.704	-0.633	0.019	-3.289	0.003	0.005
Efficiency	SMN	DAN	between	LSD	0.644	0.694	0.809	0.016	3.133	0.007	0.017
Efficiency	SMN	DAN	between	PSIL	0.746	0.815	1.186	0.022	3.137	0.020	0.036
Efficiency	SMN	DAN	between	KTM	0.724	0.744	0.624	0.009	2.161	0.054	0.077
Efficiency	SMN	DAN	between	N2O	0.717	0.724	0.159	0.012	0.615	0.548	0.548
Efficiency	SMN	DAN	between	Sleep N1	0.688	0.666	-0.642	0.006	-3.686	0.001	0.003
Efficiency	SMN	DAN	between	Sleep N2	0.692	0.624	-1.016	0.012	-5.470	0.000	0.000

bioRxiv preprint doi: https://doi.org/10.64898/2026.02.17.706398; this version posted February 18, 2026. The copyright holder for this preprint (which was not certified by peer review) is the author/funder, who has granted bioRxiv a license to display the preprint in perpetuity. It is made available under aCC-BY 4.0 International license.

Efficiency	SMN	DAN	between	PPF1.9	0.661	0.599	-1.283	0.013	-4.802	0.000	0.002
Efficiency	SMN	DAN	between	PPF2.4	0.659	0.641	-0.286	0.013	-1.459	0.157	0.177
Efficiency	SMN	DAN	between	PPF2.7	0.675	0.634	-0.379	0.021	-1.968	0.060	0.077
Efficiency	SMN	VAN	between	LSD	0.685	0.725	0.667	0.015	2.582	0.022	0.049
Efficiency	SMN	VAN	between	PSIL	0.757	0.828	2.039	0.013	5.394	0.002	0.005
Efficiency	SMN	VAN	between	KTM	0.739	0.757	0.480	0.010	1.664	0.124	0.160
Efficiency	SMN	VAN	between	N2O	0.732	0.739	0.205	0.010	0.792	0.441	0.497
Efficiency	SMN	VAN	between	Sleep N1	0.686	0.671	-0.399	0.007	-2.294	0.028	0.051
Efficiency	SMN	VAN	between	Sleep N2	0.687	0.642	-0.904	0.009	-4.868	0.000	0.000
Efficiency	SMN	VAN	between	PPF1.9	0.665	0.607	-1.208	0.013	-4.519	0.001	0.003
Efficiency	SMN	VAN	between	PPF2.4	0.673	0.667	-0.113	0.012	-0.574	0.571	0.571
Efficiency	SMN	VAN	between	PPF2.7	0.685	0.650	-0.338	0.020	-1.759	0.090	0.136
Efficiency	SMN	LIM	between	LSD	0.521	0.556	0.529	0.017	2.049	0.060	0.134
Efficiency	SMN	LIM	between	PSIL	0.624	0.659	0.265	0.050	0.702	0.509	0.764
Efficiency	SMN	LIM	between	KTM	0.639	0.669	0.674	0.013	2.336	0.039	0.118
Efficiency	SMN	LIM	between	N2O	0.595	0.614	0.301	0.016	1.166	0.263	0.474
Efficiency	SMN	LIM	between	Sleep N1	0.577	0.577	0.020	0.006	0.113	0.911	0.911
Efficiency	SMN	LIM	between	Sleep N2	0.579	0.558	-0.457	0.008	-2.460	0.020	0.091
Efficiency	SMN	LIM	between	PPF1.9	0.586	0.522	-2.035	0.008	-7.613	0.000	0.000
Efficiency	SMN	LIM	between	PPF2.4	0.597	0.592	-0.070	0.014	-0.357	0.724	0.814
Efficiency	SMN	LIM	between	PPF2.7	0.534	0.541	0.069	0.021	0.357	0.724	0.814
Efficiency	SMN	FPN	between	LSD	0.538	0.598	1.020	0.015	3.951	0.001	0.006
Efficiency	SMN	FPN	between	PSIL	0.721	0.789	0.872	0.030	2.308	0.060	0.109
Efficiency	SMN	FPN	between	KTM	0.677	0.709	0.962	0.010	3.332	0.007	0.015
Efficiency	SMN	FPN	between	N2O	0.654	0.699	0.989	0.012	3.831	0.002	0.006
Efficiency	SMN	FPN	between	Sleep N1	0.603	0.590	-0.290	0.008	-1.667	0.105	0.158
Efficiency	SMN	FPN	between	Sleep N2	0.608	0.562	-0.773	0.011	-4.164	0.000	0.002
Efficiency	SMN	FPN	between	PPF1.9	0.578	0.562	-0.270	0.015	-1.012	0.330	0.371
Efficiency	SMN	FPN	between	PPF2.4	0.573	0.597	0.302	0.016	1.542	0.136	0.174
Efficiency	SMN	FPN	between	PPF2.7	0.569	0.584	0.132	0.023	0.684	0.500	0.500
Efficiency	SMN	DMN	between	LSD	0.564	0.625	1.046	0.015	4.049	0.001	0.005
Efficiency	SMN	DMN	between	PSIL	0.742	0.789	0.536	0.033	1.419	0.206	0.309
Efficiency	SMN	DMN	between	KTM	0.675	0.703	0.749	0.011	2.596	0.025	0.056
Efficiency	SMN	DMN	between	N2O	0.683	0.706	0.506	0.012	1.960	0.070	0.126
Efficiency	SMN	DMN	between	Sleep N1	0.645	0.640	-0.186	0.005	-1.070	0.293	0.376
Efficiency	SMN	DMN	between	Sleep N2	0.647	0.614	-0.740	0.008	-3.987	0.000	0.004
Efficiency	SMN	DMN	between	PPF1.9	0.598	0.567	-0.746	0.011	-2.792	0.015	0.046
Efficiency	SMN	DMN	between	PPF2.4	0.612	0.616	0.051	0.015	0.260	0.797	0.797
Efficiency	SMN	DMN	between	PPF2.7	0.606	0.589	-0.178	0.019	-0.927	0.362	0.408
Efficiency	DAN	DAN	within	LSD	0.792	0.835	1.070	0.010	4.144	0.001	0.002
Efficiency	DAN	DAN	within	PSIL	0.865	0.910	1.193	0.014	3.156	0.020	0.025
Efficiency	DAN	DAN	within	KTM	0.837	0.842	0.117	0.013	0.407	0.692	0.692
Efficiency	DAN	DAN	within	N2O	0.844	0.839	-0.170	0.007	-0.657	0.522	0.587
Efficiency	DAN	DAN	within	Sleep N1	0.851	0.833	-0.608	0.005	-3.493	0.001	0.003
Efficiency	DAN	DAN	within	Sleep N2	0.850	0.820	-0.698	0.008	-3.760	0.001	0.002
Efficiency	DAN	DAN	within	PPF1.9	0.827	0.757	-1.405	0.013	-5.258	0.000	0.001
Efficiency	DAN	DAN	within	PPF2.4	0.831	0.798	-0.606	0.011	-3.092	0.005	0.007
Efficiency	DAN	DAN	within	PPF2.7	0.860	0.802	-0.910	0.012	-4.729	0.000	0.001
Efficiency	DAN	VAN	between	LSD	0.709	0.748	0.919	0.011	3.559	0.003	0.006
Efficiency	DAN	VAN	between	PSIL	0.815	0.852	0.744	0.019	1.967	0.097	0.124
Efficiency	DAN	VAN	between	KTM	0.747	0.768	0.474	0.013	1.640	0.129	0.145
Efficiency	DAN	VAN	between	N2O	0.787	0.775	-0.347	0.009	-1.345	0.200	0.200
Efficiency	DAN	VAN	between	Sleep N1	0.775	0.764	-0.395	0.005	-2.267	0.030	0.045
Efficiency	DAN	VAN	between	Sleep N2	0.773	0.745	-0.709	0.007	-3.818	0.001	0.002
Efficiency	DAN	VAN	between	PPF1.9	0.704	0.645	-1.159	0.014	-4.335	0.001	0.002
Efficiency	DAN	VAN	between	PPF2.4	0.762	0.726	-0.649	0.011	-3.309	0.003	0.006
Efficiency	DAN	VAN	between	PPF2.7	0.780	0.728	-0.846	0.012	-4.396	0.000	0.001
Efficiency	DAN	LIM	between	LSD	0.647	0.689	0.545	0.020	2.110	0.053	0.160
Efficiency	DAN	LIM	between	PSIL	0.744	0.742	-0.014	0.042	-0.037	0.972	0.972
Efficiency	DAN	LIM	between	KTM	0.742	0.749	0.172	0.012	0.596	0.563	0.724
Efficiency	DAN	LIM	between	N2O	0.722	0.702	-0.291	0.017	-1.127	0.279	0.493
Efficiency	DAN	LIM	between	Sleep N1	0.742	0.742	-0.006	0.006	-0.037	0.971	0.972
Efficiency	DAN	LIM	between	Sleep N2	0.738	0.748	0.251	0.008	1.350	0.188	0.423
Efficiency	DAN	LIM	between	PPF1.9	0.714	0.613	-1.000	0.027	-3.743	0.002	0.022
Efficiency	DAN	LIM	between	PPF2.4	0.757	0.710	-0.529	0.017	-2.696	0.012	0.056
Efficiency	DAN	LIM	between	PPF2.7	0.692	0.674	-0.192	0.017	-0.996	0.329	0.493
Efficiency	DAN	FPN	between	LSD	0.673	0.692	0.371	0.013	1.437	0.173	0.518

bioRxiv preprint doi: https://doi.org/10.64898/2026.02.17.706398; this version posted February 18, 2026. The copyright holder for this preprint (which was not certified by peer review) is the author/funder, who has granted bioRxiv a license to display the preprint in perpetuity. It is made available under aCC-BY 4.0 International license.

Efficiency	DAN	FPN	between	PSIL	0.791	0.816	0.421	0.022	1.114	0.308	0.554
Efficiency	DAN	FPN	between	KTM	0.725	0.728	0.073	0.013	0.252	0.806	0.907
Efficiency	DAN	FPN	between	N2O	0.739	0.736	-0.077	0.011	-0.297	0.771	0.907
Efficiency	DAN	FPN	between	Sleep N1	0.752	0.749	-0.123	0.005	-0.708	0.484	0.726
Efficiency	DAN	FPN	between	Sleep N2	0.752	0.752	-0.006	0.006	-0.034	0.973	0.973
Efficiency	DAN	FPN	between	PPF1.9	0.685	0.647	-0.625	0.016	-2.340	0.036	0.323
Efficiency	DAN	FPN	between	PPF2.4	0.728	0.713	-0.218	0.013	-1.112	0.277	0.554
Efficiency	DAN	FPN	between	PPF2.7	0.740	0.723	-0.279	0.012	-1.448	0.160	0.518
Efficiency	DAN	DMN	between	LSD	0.627	0.678	1.223	0.011	4.735	0.000	0.002
Efficiency	DAN	DMN	between	PSIL	0.766	0.803	0.336	0.042	0.888	0.409	0.526
Efficiency	DAN	DMN	between	KTM	0.694	0.721	0.488	0.016	1.689	0.119	0.215
Efficiency	DAN	DMN	between	N2O	0.706	0.714	0.161	0.012	0.622	0.544	0.612
Efficiency	DAN	DMN	between	Sleep N1	0.721	0.719	-0.058	0.006	-0.332	0.742	0.742
Efficiency	DAN	DMN	between	Sleep N2	0.719	0.699	-0.583	0.007	-3.140	0.004	0.009
Efficiency	DAN	DMN	between	PPF1.9	0.679	0.613	-1.238	0.014	-4.631	0.000	0.002
Efficiency	DAN	DMN	between	PPF2.4	0.713	0.697	-0.266	0.011	-1.357	0.187	0.280
Efficiency	DAN	DMN	between	PPF2.7	0.707	0.644	-0.669	0.018	-3.477	0.002	0.005
Efficiency	VAN	VAN	within	LSD	0.807	0.837	0.753	0.010	2.916	0.011	0.020
Efficiency	VAN	VAN	within	PSIL	0.878	0.908	1.315	0.009	3.480	0.013	0.020
Efficiency	VAN	VAN	within	KTM	0.830	0.863	0.947	0.010	3.279	0.007	0.020
Efficiency	VAN	VAN	within	N2O	0.859	0.863	0.108	0.009	0.418	0.682	0.682
Efficiency	VAN	VAN	within	Sleep N1	0.830	0.825	-0.153	0.006	-0.880	0.386	0.434
Efficiency	VAN	VAN	within	Sleep N2	0.829	0.807	-0.563	0.007	-3.030	0.005	0.020
Efficiency	VAN	VAN	within	PPF1.9	0.824	0.783	-0.792	0.014	-2.963	0.011	0.020
Efficiency	VAN	VAN	within	PPF2.4	0.852	0.845	-0.250	0.005	-1.277	0.213	0.274
Efficiency	VAN	VAN	within	PPF2.7	0.859	0.835	-0.527	0.009	-2.740	0.011	0.020
Efficiency	VAN	LIM	between	LSD	0.660	0.685	0.374	0.018	1.448	0.170	0.258
Efficiency	VAN	LIM	between	PSIL	0.767	0.728	-0.743	0.020	-1.965	0.097	0.218
Efficiency	VAN	LIM	between	KTM	0.726	0.766	0.904	0.013	3.131	0.010	0.043
Efficiency	VAN	LIM	between	N2O	0.717	0.719	0.040	0.014	0.156	0.878	0.878
Efficiency	VAN	LIM	between	Sleep N1	0.731	0.739	0.243	0.005	1.397	0.172	0.258
Efficiency	VAN	LIM	between	Sleep N2	0.733	0.732	-0.034	0.007	-0.186	0.854	0.878
Efficiency	VAN	LIM	between	PPF1.9	0.734	0.680	-0.632	0.023	-2.365	0.034	0.103
Efficiency	VAN	LIM	between	PPF2.4	0.766	0.773	0.155	0.010	0.790	0.437	0.562
Efficiency	VAN	LIM	between	PPF2.7	0.686	0.738	0.610	0.017	3.168	0.004	0.035
Efficiency	VAN	FPN	between	LSD	0.692	0.724	0.656	0.012	2.539	0.024	0.054
Efficiency	VAN	FPN	between	PSIL	0.811	0.822	0.194	0.020	0.514	0.626	0.626
Efficiency	VAN	FPN	between	KTM	0.756	0.783	0.794	0.010	2.750	0.019	0.054
Efficiency	VAN	FPN	between	N2O	0.779	0.770	-0.214	0.011	-0.830	0.420	0.626
Efficiency	VAN	FPN	between	Sleep N1	0.772	0.775	0.105	0.004	0.604	0.550	0.626
Efficiency	VAN	FPN	between	Sleep N2	0.772	0.759	-0.443	0.005	-2.388	0.024	0.054
Efficiency	VAN	FPN	between	PPF1.9	0.715	0.677	-0.967	0.010	-3.617	0.003	0.028
Efficiency	VAN	FPN	between	PPF2.4	0.770	0.764	-0.115	0.010	-0.589	0.561	0.626
Efficiency	VAN	FPN	between	PPF2.7	0.759	0.745	-0.256	0.011	-1.332	0.195	0.350
Efficiency	VAN	DMN	between	LSD	0.660	0.700	0.989	0.011	3.830	0.002	0.007
Efficiency	VAN	DMN	between	PSIL	0.783	0.801	0.199	0.035	0.527	0.617	0.617
Efficiency	VAN	DMN	between	KTM	0.706	0.735	0.758	0.011	2.626	0.024	0.053
Efficiency	VAN	DMN	between	N2O	0.734	0.723	-0.264	0.011	-1.022	0.324	0.486
Efficiency	VAN	DMN	between	Sleep N1	0.723	0.730	0.239	0.005	1.373	0.179	0.323
Efficiency	VAN	DMN	between	Sleep N2	0.720	0.714	-0.162	0.007	-0.870	0.392	0.504
Efficiency	VAN	DMN	between	PPF1.9	0.693	0.635	-1.004	0.015	-3.756	0.002	0.007
Efficiency	VAN	DMN	between	PPF2.4	0.739	0.734	-0.133	0.008	-0.679	0.503	0.566
Efficiency	VAN	DMN	between	PPF2.7	0.718	0.662	-0.803	0.013	-4.172	0.000	0.003
Efficiency	LIM	LIM	within	LSD	0.893	0.894	0.030	0.011	0.117	0.908	0.908
Efficiency	LIM	LIM	within	PSIL	0.955	0.949	-0.244	0.008	-0.645	0.543	0.682
Efficiency	LIM	LIM	within	KTM	0.849	0.840	-0.191	0.013	-0.663	0.521	0.682
Efficiency	LIM	LIM	within	N2O	0.894	0.872	-0.493	0.011	-1.910	0.077	0.346
Efficiency	LIM	LIM	within	Sleep N1	0.862	0.858	-0.091	0.008	-0.521	0.606	0.682
Efficiency	LIM	LIM	within	Sleep N2	0.863	0.850	-0.290	0.008	-1.562	0.130	0.389
Efficiency	LIM	LIM	within	PPF1.9	0.872	0.844	-0.278	0.027	-1.040	0.317	0.571
Efficiency	LIM	LIM	within	PPF2.4	0.883	0.872	-0.213	0.011	-1.089	0.287	0.571
Efficiency	LIM	LIM	within	PPF2.7	0.825	0.882	0.583	0.019	3.030	0.005	0.049
Efficiency	LIM	FPN	between	LSD	0.681	0.704	0.352	0.017	1.362	0.195	0.292
Efficiency	LIM	FPN	between	PSIL	0.777	0.722	-0.644	0.032	-1.704	0.139	0.251
Efficiency	LIM	FPN	between	KTM	0.740	0.748	0.195	0.011	0.675	0.514	0.578
Efficiency	LIM	FPN	between	N2O	0.728	0.715	-0.244	0.014	-0.946	0.360	0.463
Efficiency	LIM	FPN	between	Sleep N1	0.757	0.772	0.498	0.005	2.859	0.007	0.022

bioRxiv preprint doi: https://doi.org/10.64898/2026.02.17.706398; this version posted February 18, 2026. The copyright holder for this preprint (which was not certified by peer review) is the author/funder, who has granted bioRxiv a license to display the preprint in perpetuity. It is made available under aCC-BY 4.0 International license.

Efficiency	LIM	FPN	between	Sleep N2	0.757	0.773	0.507	0.006	2.730	0.011	0.024
Efficiency	LIM	FPN	between	PPF1.9	0.734	0.641	-0.933	0.027	-3.491	0.004	0.018
Efficiency	LIM	FPN	between	PPF2.4	0.788	0.751	-0.663	0.011	-3.382	0.002	0.018
Efficiency	LIM	FPN	between	PPF2.7	0.706	0.702	-0.038	0.016	-0.197	0.845	0.845
Efficiency	LIM	DMN	between	LSD	0.641	0.677	0.616	0.015	2.387	0.032	0.063
Efficiency	LIM	DMN	between	PSIL	0.748	0.694	-0.389	0.052	-1.029	0.343	0.386
Efficiency	LIM	DMN	between	KTM	0.663	0.687	0.425	0.016	1.472	0.169	0.217
Efficiency	LIM	DMN	between	N2O	0.688	0.656	-0.529	0.016	-2.047	0.060	0.090
Efficiency	LIM	DMN	between	Sleep N1	0.681	0.698	0.452	0.007	2.598	0.014	0.042
Efficiency	LIM	DMN	between	Sleep N2	0.676	0.680	0.074	0.009	0.399	0.693	0.693
Efficiency	LIM	DMN	between	PPF1.9	0.681	0.567	-1.135	0.027	-4.249	0.001	0.006
Efficiency	LIM	DMN	between	PPF2.4	0.743	0.701	-0.706	0.012	-3.601	0.001	0.006
Efficiency	LIM	DMN	between	PPF2.7	0.651	0.611	-0.428	0.018	-2.226	0.035	0.063
Efficiency	FPN	FPN	within	LSD	0.812	0.835	0.604	0.010	2.338	0.035	0.078
Efficiency	FPN	FPN	within	PSIL	0.872	0.883	0.226	0.019	0.598	0.572	0.643
Efficiency	FPN	FPN	within	KTM	0.837	0.852	0.458	0.009	1.588	0.141	0.253
Efficiency	FPN	FPN	within	N2O	0.828	0.815	-0.347	0.010	-1.345	0.200	0.300
Efficiency	FPN	FPN	within	Sleep N1	0.851	0.849	-0.033	0.006	-0.191	0.850	0.850
Efficiency	FPN	FPN	within	Sleep N2	0.850	0.845	-0.164	0.006	-0.883	0.385	0.495
Efficiency	FPN	FPN	within	PPF1.9	0.832	0.776	-1.052	0.014	-3.936	0.002	0.015
Efficiency	FPN	FPN	within	PPF2.4	0.848	0.824	-0.540	0.009	-2.755	0.011	0.032
Efficiency	FPN	FPN	within	PPF2.7	0.837	0.810	-0.596	0.009	-3.098	0.005	0.021
Efficiency	FPN	DMN	between	LSD	0.688	0.722	0.747	0.012	2.892	0.012	0.035
Efficiency	FPN	DMN	between	PSIL	0.789	0.794	0.067	0.031	0.177	0.865	0.865
Efficiency	FPN	DMN	between	KTM	0.721	0.741	0.435	0.013	1.505	0.160	0.291
Efficiency	FPN	DMN	between	N2O	0.731	0.727	-0.084	0.011	-0.324	0.751	0.844
Efficiency	FPN	DMN	between	Sleep N1	0.739	0.743	0.097	0.006	0.557	0.581	0.747
Efficiency	FPN	DMN	between	Sleep N2	0.737	0.725	-0.267	0.009	-1.438	0.162	0.291
Efficiency	FPN	DMN	between	PPF1.9	0.700	0.646	-0.865	0.017	-3.235	0.007	0.029
Efficiency	FPN	DMN	between	PPF2.4	0.749	0.742	-0.157	0.009	-0.798	0.432	0.648
Efficiency	FPN	DMN	between	PPF2.7	0.726	0.679	-0.669	0.013	-3.476	0.002	0.016
Efficiency	DMN	DMN	within	LSD	0.767	0.805	1.322	0.007	5.119	0.000	0.001
Efficiency	DMN	DMN	within	PSIL	0.847	0.859	0.167	0.026	0.443	0.673	0.758
Efficiency	DMN	DMN	within	KTM	0.789	0.804	0.383	0.011	1.328	0.211	0.363
Efficiency	DMN	DMN	within	N2O	0.795	0.781	-0.316	0.011	-1.222	0.242	0.363
Efficiency	DMN	DMN	within	Sleep N1	0.804	0.801	-0.080	0.006	-0.458	0.650	0.758
Efficiency	DMN	DMN	within	Sleep N2	0.800	0.782	-0.436	0.008	-2.348	0.026	0.059
Efficiency	DMN	DMN	within	PPF1.9	0.784	0.718	-1.016	0.017	-3.801	0.002	0.007
Efficiency	DMN	DMN	within	PPF2.4	0.786	0.785	-0.011	0.006	-0.058	0.954	0.954
Efficiency	DMN	DMN	within	PPF2.7	0.789	0.736	-0.880	0.012	-4.575	0.000	0.001
Complexity	VIS	VIS	within	LSD	0.240	0.242	0.304	0.002	1.176	0.259	0.291
Complexity	VIS	VIS	within	PSIL	0.253	0.256	0.319	0.004	0.844	0.431	0.431
Complexity	VIS	VIS	within	KTM	0.291	0.281	-0.973	0.003	-3.371	0.006	0.011
Complexity	VIS	VIS	within	N2O	0.270	0.265	-0.361	0.004	-1.397	0.184	0.237
Complexity	VIS	VIS	within	Sleep N1	0.268	0.266	-0.240	0.001	-1.379	0.177	0.237
Complexity	VIS	VIS	within	Sleep N2	0.268	0.260	-0.841	0.002	-4.529	0.000	0.000
Complexity	VIS	VIS	within	PPF1.9	0.274	0.266	-0.994	0.002	-3.718	0.003	0.008
Complexity	VIS	VIS	within	PPF2.4	0.179	0.165	-1.461	0.002	-7.451	0.000	0.000
Complexity	VIS	VIS	within	PPF2.7	0.228	0.219	-0.622	0.003	-3.229	0.003	0.008
Complexity	VIS	SMN	between	LSD	0.227	0.232	0.427	0.003	1.653	0.121	0.181
Complexity	VIS	SMN	between	PSIL	0.244	0.250	0.535	0.004	1.414	0.207	0.266
Complexity	VIS	SMN	between	KTM	0.280	0.278	-0.111	0.003	-0.384	0.708	0.708
Complexity	VIS	SMN	between	N2O	0.258	0.254	-0.238	0.004	-0.921	0.373	0.419
Complexity	VIS	SMN	between	Sleep N1	0.257	0.254	-0.388	0.001	-2.231	0.033	0.059
Complexity	VIS	SMN	between	Sleep N2	0.257	0.249	-0.790	0.002	-4.255	0.000	0.001
Complexity	VIS	SMN	between	PPF1.9	0.265	0.251	-1.293	0.003	-4.839	0.000	0.001
Complexity	VIS	SMN	between	PPF2.4	0.167	0.149	-1.733	0.002	-8.839	0.000	0.000
Complexity	VIS	SMN	between	PPF2.7	0.216	0.201	-0.971	0.003	-5.044	0.000	0.000
Complexity	VIS	DAN	between	LSD	0.236	0.246	0.981	0.003	3.798	0.002	0.004
Complexity	VIS	DAN	between	PSIL	0.260	0.267	0.466	0.006	1.232	0.264	0.339
Complexity	VIS	DAN	between	KTM	0.294	0.294	0.018	0.004	0.061	0.952	0.952
Complexity	VIS	DAN	between	N2O	0.278	0.274	-0.232	0.004	-0.897	0.385	0.433
Complexity	VIS	DAN	between	Sleep N1	0.276	0.274	-0.210	0.002	-1.208	0.236	0.339
Complexity	VIS	DAN	between	Sleep N2	0.276	0.266	-0.689	0.003	-3.713	0.001	0.003
Complexity	VIS	DAN	between	PPF1.9	0.280	0.267	-1.102	0.003	-4.122	0.001	0.003
Complexity	VIS	DAN	between	PPF2.4	0.174	0.159	-1.331	0.002	-6.785	0.000	0.000
Complexity	VIS	DAN	between	PPF2.7	0.228	0.217	-0.817	0.003	-4.247	0.000	0.001

bioRxiv preprint doi: https://doi.org/10.64898/2026.02.17.706398; this version posted February 18, 2026. The copyright holder for this preprint (which was not certified by peer review) is the author/funder, who has granted bioRxiv a license to display the preprint in perpetuity. It is made available under aCC-BY 4.0 International license.

Complexity	VIS	VAN	between	LSD	0.239	0.245	0.658	0.002	2.548	0.023	0.042
Complexity	VIS	VAN	between	PSIL	0.258	0.265	0.499	0.006	1.320	0.235	0.302
Complexity	VIS	VAN	between	KTM	0.293	0.291	-0.256	0.003	-0.887	0.394	0.444
Complexity	VIS	VAN	between	N2O	0.277	0.274	-0.189	0.005	-0.733	0.476	0.476
Complexity	VIS	VAN	between	Sleep N1	0.272	0.270	-0.261	0.002	-1.501	0.143	0.215
Complexity	VIS	VAN	between	Sleep N2	0.272	0.265	-0.601	0.002	-3.238	0.003	0.007
Complexity	VIS	VAN	between	PPF1.9	0.280	0.269	-1.011	0.003	-3.783	0.002	0.007
Complexity	VIS	VAN	between	PPF2.4	0.169	0.156	-1.272	0.002	-6.485	0.000	0.000
Complexity	VIS	VAN	between	PPF2.7	0.223	0.211	-1.131	0.002	-5.875	0.000	0.000
Complexity	VIS	LIM	between	LSD	0.280	0.284	0.291	0.003	1.126	0.279	0.365
Complexity	VIS	LIM	between	PSIL	0.306	0.313	0.391	0.007	1.034	0.341	0.384
Complexity	VIS	LIM	between	KTM	0.343	0.339	-0.390	0.003	-1.352	0.204	0.365
Complexity	VIS	LIM	between	N2O	0.322	0.314	-0.401	0.005	-1.553	0.143	0.321
Complexity	VIS	LIM	between	Sleep N1	0.319	0.321	0.138	0.002	0.792	0.434	0.434
Complexity	VIS	LIM	between	Sleep N2	0.319	0.316	-0.203	0.002	-1.093	0.284	0.365
Complexity	VIS	LIM	between	PPF1.9	0.330	0.317	-0.566	0.006	-2.119	0.054	0.162
Complexity	VIS	LIM	between	PPF2.4	0.199	0.183	-1.568	0.002	-7.995	0.000	0.000
Complexity	VIS	LIM	between	PPF2.7	0.257	0.250	-0.430	0.003	-2.232	0.034	0.155
Complexity	VIS	FPN	between	LSD	0.225	0.232	0.680	0.003	2.632	0.020	0.059
Complexity	VIS	FPN	between	PSIL	0.250	0.259	0.549	0.006	1.454	0.196	0.294
Complexity	VIS	FPN	between	KTM	0.280	0.280	-0.050	0.003	-0.172	0.866	0.975
Complexity	VIS	FPN	between	N2O	0.267	0.267	0.002	0.005	0.009	0.993	0.993
Complexity	VIS	FPN	between	Sleep N1	0.261	0.261	-0.032	0.001	-0.184	0.855	0.975
Complexity	VIS	FPN	between	Sleep N2	0.261	0.256	-0.525	0.002	-2.829	0.009	0.038
Complexity	VIS	FPN	between	PPF1.9	0.268	0.260	-0.654	0.003	-2.446	0.029	0.066
Complexity	VIS	FPN	between	PPF2.4	0.160	0.151	-0.887	0.002	-4.524	0.000	0.001
Complexity	VIS	FPN	between	PPF2.7	0.211	0.205	-0.420	0.002	-2.181	0.038	0.069
Complexity	VIS	DMN	between	LSD	0.218	0.224	0.523	0.003	2.025	0.062	0.112
Complexity	VIS	DMN	between	PSIL	0.241	0.250	0.552	0.006	1.460	0.194	0.290
Complexity	VIS	DMN	between	KTM	0.271	0.271	-0.067	0.004	-0.234	0.819	0.819
Complexity	VIS	DMN	between	N2O	0.262	0.257	-0.327	0.004	-1.268	0.226	0.290
Complexity	VIS	DMN	between	Sleep N1	0.255	0.254	-0.124	0.001	-0.711	0.482	0.543
Complexity	VIS	DMN	between	Sleep N2	0.255	0.246	-0.897	0.002	-4.829	0.000	0.000
Complexity	VIS	DMN	between	PPF1.9	0.259	0.249	-1.136	0.002	-4.251	0.001	0.002
Complexity	VIS	DMN	between	PPF2.4	0.155	0.144	-1.011	0.002	-5.155	0.000	0.000
Complexity	VIS	DMN	between	PPF2.7	0.205	0.197	-0.763	0.002	-3.966	0.001	0.002
Complexity	SMN	SMN	within	LSD	0.236	0.236	0.037	0.003	0.145	0.887	0.887
Complexity	SMN	SMN	within	PSIL	0.242	0.244	0.276	0.003	0.731	0.492	0.686
Complexity	SMN	SMN	within	KTM	0.281	0.282	0.114	0.002	0.393	0.702	0.789
Complexity	SMN	SMN	within	N2O	0.261	0.259	-0.243	0.003	-0.942	0.362	0.652
Complexity	SMN	SMN	within	Sleep N1	0.258	0.257	-0.110	0.001	-0.630	0.533	0.686
Complexity	SMN	SMN	within	Sleep N2	0.257	0.252	-0.433	0.002	-2.334	0.027	0.061
Complexity	SMN	SMN	within	PPF1.9	0.264	0.253	-0.883	0.003	-3.303	0.006	0.017
Complexity	SMN	SMN	within	PPF2.4	0.172	0.158	-1.577	0.002	-8.040	0.000	0.000
Complexity	SMN	SMN	within	PPF2.7	0.217	0.204	-1.161	0.002	-6.033	0.000	0.000
Complexity	SMN	DAN	between	LSD	0.241	0.250	0.506	0.004	1.960	0.070	0.126
Complexity	SMN	DAN	between	PSIL	0.262	0.269	0.610	0.005	1.613	0.158	0.203
Complexity	SMN	DAN	between	KTM	0.302	0.307	0.454	0.003	1.571	0.144	0.203
Complexity	SMN	DAN	between	N2O	0.283	0.286	0.187	0.004	0.723	0.482	0.482
Complexity	SMN	DAN	between	Sleep N1	0.281	0.278	-0.229	0.002	-1.313	0.199	0.223
Complexity	SMN	DAN	between	Sleep N2	0.281	0.271	-0.608	0.003	-3.276	0.003	0.006
Complexity	SMN	DAN	between	PPF1.9	0.283	0.267	-1.085	0.004	-4.059	0.001	0.004
Complexity	SMN	DAN	between	PPF2.4	0.175	0.159	-1.436	0.002	-7.320	0.000	0.000
Complexity	SMN	DAN	between	PPF2.7	0.229	0.212	-1.092	0.003	-5.674	0.000	0.000
Complexity	SMN	VAN	between	LSD	0.247	0.250	0.199	0.003	0.769	0.455	0.585
Complexity	SMN	VAN	between	PSIL	0.263	0.270	0.556	0.005	1.470	0.192	0.288
Complexity	SMN	VAN	between	KTM	0.304	0.309	0.473	0.003	1.640	0.129	0.233
Complexity	SMN	VAN	between	N2O	0.287	0.289	0.118	0.003	0.457	0.655	0.660
Complexity	SMN	VAN	between	Sleep N1	0.282	0.281	-0.077	0.002	-0.444	0.660	0.660
Complexity	SMN	VAN	between	Sleep N2	0.282	0.275	-0.610	0.002	-3.284	0.003	0.008
Complexity	SMN	VAN	between	PPF1.9	0.287	0.276	-0.662	0.004	-2.476	0.028	0.063
Complexity	SMN	VAN	between	PPF2.4	0.175	0.162	-1.186	0.002	-6.047	0.000	0.000
Complexity	SMN	VAN	between	PPF2.7	0.229	0.215	-1.254	0.002	-6.516	0.000	0.000
Complexity	SMN	LIM	between	LSD	0.280	0.282	0.117	0.004	0.453	0.658	0.920
Complexity	SMN	LIM	between	PSIL	0.310	0.309	-0.040	0.004	-0.105	0.920	0.920
Complexity	SMN	LIM	between	KTM	0.348	0.348	-0.032	0.002	-0.112	0.913	0.920
Complexity	SMN	LIM	between	N2O	0.320	0.315	-0.243	0.005	-0.941	0.363	0.653

bioRxiv preprint doi: https://doi.org/10.64898/2026.02.17.706398; this version posted February 18, 2026. The copyright holder for this preprint (which was not certified by peer review) is the author/funder, who has granted bioRxiv a license to display the preprint in perpetuity. It is made available under aCC-BY 4.0 International license.

Complexity	SMN	LIM	between	Sleep N1	0.322	0.322	0.032	0.002	0.182	0.857	0.920
Complexity	SMN	LIM	between	Sleep N2	0.322	0.319	-0.210	0.003	-1.132	0.267	0.602
Complexity	SMN	LIM	between	PPF1.9	0.334	0.314	-0.764	0.007	-2.859	0.013	0.060
Complexity	SMN	LIM	between	PPF2.4	0.199	0.183	-1.392	0.002	-7.096	0.000	0.000
Complexity	SMN	LIM	between	PPF2.7	0.255	0.247	-0.426	0.003	-2.214	0.036	0.108
Complexity	SMN	FPN	between	LSD	0.226	0.234	0.679	0.003	2.630	0.020	0.036
Complexity	SMN	FPN	between	PSIL	0.253	0.260	0.549	0.005	1.453	0.196	0.253
Complexity	SMN	FPN	between	KTM	0.285	0.290	0.445	0.003	1.541	0.152	0.227
Complexity	SMN	FPN	between	N2O	0.270	0.273	0.272	0.004	1.055	0.309	0.348
Complexity	SMN	FPN	between	Sleep N1	0.266	0.266	-0.022	0.002	-0.127	0.900	0.900
Complexity	SMN	FPN	between	Sleep N2	0.266	0.260	-0.538	0.002	-2.896	0.007	0.022
Complexity	SMN	FPN	between	PPF1.9	0.271	0.260	-0.726	0.004	-2.716	0.018	0.036
Complexity	SMN	FPN	between	PPF2.4	0.161	0.151	-0.903	0.002	-4.605	0.000	0.001
Complexity	SMN	FPN	between	PPF2.7	0.211	0.204	-0.601	0.002	-3.122	0.004	0.020
Complexity	SMN	DMN	between	LSD	0.208	0.211	0.279	0.003	1.081	0.298	0.383
Complexity	SMN	DMN	between	PSIL	0.230	0.237	0.506	0.005	1.340	0.229	0.343
Complexity	SMN	DMN	between	KTM	0.260	0.264	0.375	0.003	1.297	0.221	0.343
Complexity	SMN	DMN	between	N2O	0.245	0.247	0.114	0.004	0.442	0.665	0.748
Complexity	SMN	DMN	between	Sleep N1	0.243	0.243	-0.026	0.002	-0.148	0.883	0.883
Complexity	SMN	DMN	between	Sleep N2	0.244	0.237	-0.646	0.002	-3.481	0.002	0.005
Complexity	SMN	DMN	between	PPF1.9	0.246	0.236	-0.771	0.003	-2.883	0.013	0.029
Complexity	SMN	DMN	between	PPF2.4	0.147	0.137	-0.986	0.002	-5.029	0.000	0.000
Complexity	SMN	DMN	between	PPF2.7	0.193	0.185	-0.806	0.002	-4.188	0.000	0.001
Complexity	DAN	DAN	within	LSD	0.255	0.261	0.543	0.003	2.104	0.054	0.097
Complexity	DAN	DAN	within	PSIL	0.269	0.273	0.375	0.004	0.992	0.359	0.539
Complexity	DAN	DAN	within	KTM	0.309	0.310	0.151	0.003	0.524	0.610	0.687
Complexity	DAN	DAN	within	N2O	0.293	0.291	-0.104	0.004	-0.404	0.692	0.692
Complexity	DAN	DAN	within	Sleep N1	0.290	0.289	-0.109	0.001	-0.627	0.535	0.687
Complexity	DAN	DAN	within	Sleep N2	0.291	0.285	-0.445	0.002	-2.399	0.023	0.053
Complexity	DAN	DAN	within	PPF1.9	0.292	0.277	-1.399	0.003	-5.236	0.000	0.000
Complexity	DAN	DAN	within	PPF2.4	0.187	0.172	-1.415	0.002	-7.214	0.000	0.000
Complexity	DAN	DAN	within	PPF2.7	0.241	0.230	-0.881	0.002	-4.577	0.000	0.000
Complexity	DAN	VAN	between	LSD	0.242	0.247	0.396	0.003	1.534	0.147	0.201
Complexity	DAN	VAN	between	PSIL	0.261	0.269	0.518	0.005	1.371	0.220	0.247
Complexity	DAN	VAN	between	KTM	0.300	0.304	0.440	0.003	1.523	0.156	0.201
Complexity	DAN	VAN	between	N2O	0.286	0.287	0.065	0.004	0.250	0.806	0.806
Complexity	DAN	VAN	between	Sleep N1	0.282	0.279	-0.314	0.001	-1.805	0.081	0.145
Complexity	DAN	VAN	between	Sleep N2	0.282	0.273	-0.748	0.002	-4.030	0.000	0.001
Complexity	DAN	VAN	between	PPF1.9	0.285	0.271	-0.988	0.004	-3.696	0.003	0.006
Complexity	DAN	VAN	between	PPF2.4	0.172	0.158	-1.252	0.002	-6.382	0.000	0.000
Complexity	DAN	VAN	between	PPF2.7	0.228	0.213	-1.364	0.002	-7.090	0.000	0.000
Complexity	DAN	LIM	between	LSD	0.276	0.281	0.308	0.003	1.194	0.252	0.568
Complexity	DAN	LIM	between	PSIL	0.304	0.306	0.113	0.006	0.299	0.775	0.775
Complexity	DAN	LIM	between	KTM	0.345	0.344	-0.103	0.002	-0.358	0.727	0.775
Complexity	DAN	LIM	between	N2O	0.320	0.315	-0.208	0.005	-0.804	0.435	0.652
Complexity	DAN	LIM	between	Sleep N1	0.325	0.326	0.097	0.002	0.556	0.582	0.749
Complexity	DAN	LIM	between	Sleep N2	0.325	0.323	-0.169	0.002	-0.912	0.370	0.652
Complexity	DAN	LIM	between	PPF1.9	0.331	0.311	-0.806	0.007	-3.016	0.010	0.030
Complexity	DAN	LIM	between	PPF2.4	0.197	0.181	-1.504	0.002	-7.669	0.000	0.000
Complexity	DAN	LIM	between	PPF2.7	0.259	0.248	-0.771	0.003	-4.007	0.000	0.002
Complexity	DAN	FPN	between	LSD	0.230	0.239	0.705	0.003	2.731	0.016	0.037
Complexity	DAN	FPN	between	PSIL	0.261	0.266	0.335	0.006	0.888	0.409	0.526
Complexity	DAN	FPN	between	KTM	0.294	0.298	0.389	0.003	1.347	0.205	0.308
Complexity	DAN	FPN	between	N2O	0.282	0.283	0.071	0.004	0.274	0.788	0.788
Complexity	DAN	FPN	between	Sleep N1	0.279	0.279	0.108	0.001	0.623	0.538	0.605
Complexity	DAN	FPN	between	Sleep N2	0.279	0.275	-0.393	0.002	-2.116	0.043	0.078
Complexity	DAN	FPN	between	PPF1.9	0.280	0.269	-0.810	0.004	-3.031	0.010	0.029
Complexity	DAN	FPN	between	PPF2.4	0.168	0.159	-0.898	0.002	-4.579	0.000	0.001
Complexity	DAN	FPN	between	PPF2.7	0.224	0.215	-0.728	0.002	-3.784	0.001	0.004
Complexity	DAN	DMN	between	LSD	0.233	0.237	0.376	0.003	1.458	0.167	0.256
Complexity	DAN	DMN	between	PSIL	0.255	0.266	0.580	0.007	1.535	0.176	0.256
Complexity	DAN	DMN	between	KTM	0.291	0.295	0.394	0.004	1.367	0.199	0.256
Complexity	DAN	DMN	between	N2O	0.278	0.279	0.085	0.004	0.329	0.747	0.760
Complexity	DAN	DMN	between	Sleep N1	0.276	0.277	0.054	0.002	0.308	0.760	0.760
Complexity	DAN	DMN	between	Sleep N2	0.276	0.269	-0.535	0.003	-2.883	0.007	0.017
Complexity	DAN	DMN	between	PPF1.9	0.276	0.263	-0.927	0.004	-3.470	0.004	0.012
Complexity	DAN	DMN	between	PPF2.4	0.164	0.154	-1.089	0.002	-5.552	0.000	0.000

bioRxiv preprint doi: https://doi.org/10.64898/2026.02.17.706398; this version posted February 18, 2026. The copyright holder for this preprint (which was not certified by peer review) is the author/funder, who has granted bioRxiv a license to display the preprint in perpetuity. It is made available under aCC-BY 4.0 International license.

Complexity	DAN	DMN	between	PPF2.7	0.221	0.210	-0.937	0.002	-4.868	0.000	0.000
Complexity	VAN	VAN	within	LSD	0.262	0.262	0.032	0.003	0.122	0.904	0.904
Complexity	VAN	VAN	within	PSIL	0.269	0.273	0.375	0.004	0.993	0.359	0.646
Complexity	VAN	VAN	within	KTM	0.312	0.312	0.074	0.002	0.256	0.803	0.903
Complexity	VAN	VAN	within	N2O	0.296	0.294	-0.104	0.005	-0.404	0.692	0.890
Complexity	VAN	VAN	within	Sleep N1	0.293	0.292	-0.136	0.001	-0.781	0.441	0.661
Complexity	VAN	VAN	within	Sleep N2	0.293	0.287	-0.464	0.002	-2.499	0.019	0.042
Complexity	VAN	VAN	within	PPF1.9	0.301	0.288	-0.898	0.004	-3.358	0.005	0.015
Complexity	VAN	VAN	within	PPF2.4	0.186	0.172	-1.275	0.002	-6.503	0.000	0.000
Complexity	VAN	VAN	within	PPF2.7	0.243	0.226	-1.806	0.002	-9.383	0.000	0.000
Complexity	VAN	LIM	between	LSD	0.277	0.280	0.250	0.002	0.969	0.349	0.523
Complexity	VAN	LIM	between	PSIL	0.305	0.307	0.133	0.005	0.353	0.737	0.829
Complexity	VAN	LIM	between	KTM	0.349	0.345	-0.434	0.002	-1.502	0.161	0.363
Complexity	VAN	LIM	between	N2O	0.319	0.316	-0.155	0.005	-0.600	0.558	0.718
Complexity	VAN	LIM	between	Sleep N1	0.326	0.326	-0.001	0.002	-0.008	0.994	0.994
Complexity	VAN	LIM	between	Sleep N2	0.326	0.323	-0.231	0.002	-1.243	0.224	0.403
Complexity	VAN	LIM	between	PPF1.9	0.339	0.321	-0.699	0.007	-2.617	0.021	0.064
Complexity	VAN	LIM	between	PPF2.4	0.196	0.182	-1.230	0.002	-6.272	0.000	0.000
Complexity	VAN	LIM	between	PPF2.7	0.261	0.250	-0.813	0.003	-4.223	0.000	0.001
Complexity	VAN	FPN	between	LSD	0.232	0.237	0.506	0.003	1.961	0.070	0.126
Complexity	VAN	FPN	between	PSIL	0.258	0.264	0.348	0.007	0.922	0.392	0.504
Complexity	VAN	FPN	between	KTM	0.294	0.300	0.521	0.003	1.805	0.099	0.148
Complexity	VAN	FPN	between	N2O	0.282	0.284	0.100	0.004	0.386	0.705	0.794
Complexity	VAN	FPN	between	Sleep N1	0.279	0.279	0.033	0.001	0.190	0.850	0.850
Complexity	VAN	FPN	between	Sleep N2	0.279	0.272	-0.629	0.002	-3.388	0.002	0.006
Complexity	VAN	FPN	between	PPF1.9	0.285	0.275	-0.726	0.004	-2.718	0.018	0.040
Complexity	VAN	FPN	between	PPF2.4	0.167	0.157	-0.978	0.002	-4.986	0.000	0.000
Complexity	VAN	FPN	between	PPF2.7	0.222	0.211	-1.103	0.002	-5.729	0.000	0.000
Complexity	VAN	DMN	between	LSD	0.231	0.236	0.522	0.003	2.023	0.063	0.113
Complexity	VAN	DMN	between	PSIL	0.255	0.265	0.541	0.007	1.430	0.203	0.260
Complexity	VAN	DMN	between	KTM	0.292	0.297	0.539	0.003	1.866	0.089	0.133
Complexity	VAN	DMN	between	N2O	0.276	0.278	0.123	0.005	0.475	0.642	0.661
Complexity	VAN	DMN	between	Sleep N1	0.276	0.277	0.077	0.001	0.443	0.661	0.661
Complexity	VAN	DMN	between	Sleep N2	0.277	0.270	-0.597	0.002	-3.217	0.003	0.010
Complexity	VAN	DMN	between	PPF1.9	0.283	0.275	-0.556	0.004	-2.080	0.058	0.113
Complexity	VAN	DMN	between	PPF2.4	0.165	0.155	-0.938	0.002	-4.782	0.000	0.000
Complexity	VAN	DMN	between	PPF2.7	0.221	0.210	-1.134	0.002	-5.895	0.000	0.000
Complexity	LIM	LIM	within	LSD	0.298	0.298	-0.007	0.005	-0.028	0.978	0.978
Complexity	LIM	LIM	within	PSIL	0.325	0.327	0.241	0.003	0.637	0.548	0.616
Complexity	LIM	LIM	within	KTM	0.358	0.353	-0.307	0.004	-1.062	0.311	0.591
Complexity	LIM	LIM	within	N2O	0.343	0.339	-0.261	0.004	-1.013	0.328	0.591
Complexity	LIM	LIM	within	Sleep N1	0.340	0.342	0.133	0.002	0.763	0.451	0.616
Complexity	LIM	LIM	within	Sleep N2	0.340	0.341	0.131	0.003	0.703	0.488	0.616
Complexity	LIM	LIM	within	PPF1.9	0.350	0.338	-0.407	0.008	-1.525	0.151	0.454
Complexity	LIM	LIM	within	PPF2.4	0.213	0.200	-1.089	0.002	-5.555	0.000	0.000
Complexity	LIM	LIM	within	PPF2.7	0.277	0.267	-0.627	0.003	-3.259	0.003	0.014
Complexity	LIM	FPN	between	LSD	0.273	0.278	0.293	0.004	1.136	0.275	0.495
Complexity	LIM	FPN	between	PSIL	0.307	0.310	0.232	0.005	0.613	0.562	0.651
Complexity	LIM	FPN	between	KTM	0.342	0.343	0.126	0.004	0.437	0.671	0.671
Complexity	LIM	FPN	between	N2O	0.324	0.319	-0.303	0.004	-1.175	0.260	0.495
Complexity	LIM	FPN	between	Sleep N1	0.327	0.328	0.098	0.002	0.561	0.578	0.651
Complexity	LIM	FPN	between	Sleep N2	0.327	0.326	-0.107	0.002	-0.579	0.567	0.651
Complexity	LIM	FPN	between	PPF1.9	0.339	0.320	-0.669	0.007	-2.502	0.026	0.079
Complexity	LIM	FPN	between	PPF2.4	0.194	0.181	-1.399	0.002	-7.134	0.000	0.000
Complexity	LIM	FPN	between	PPF2.7	0.262	0.250	-0.876	0.002	-4.553	0.000	0.000
Complexity	LIM	DMN	between	LSD	0.278	0.284	0.342	0.005	1.325	0.207	0.310
Complexity	LIM	DMN	between	PSIL	0.313	0.316	0.225	0.004	0.595	0.574	0.590
Complexity	LIM	DMN	between	KTM	0.343	0.345	0.160	0.004	0.555	0.590	0.590
Complexity	LIM	DMN	between	N2O	0.330	0.319	-0.539	0.005	-2.086	0.056	0.126
Complexity	LIM	DMN	between	Sleep N1	0.330	0.331	0.100	0.002	0.572	0.571	0.590
Complexity	LIM	DMN	between	Sleep N2	0.330	0.326	-0.260	0.002	-1.399	0.173	0.310
Complexity	LIM	DMN	between	PPF1.9	0.341	0.320	-0.775	0.007	-2.901	0.012	0.037
Complexity	LIM	DMN	between	PPF2.4	0.199	0.182	-1.646	0.002	-8.394	0.000	0.000
Complexity	LIM	DMN	between	PPF2.7	0.265	0.252	-0.887	0.003	-4.607	0.000	0.000
Complexity	FPN	FPN	within	LSD	0.239	0.247	0.666	0.003	2.580	0.022	0.039
Complexity	FPN	FPN	within	PSIL	0.260	0.264	0.328	0.005	0.867	0.419	0.626
Complexity	FPN	FPN	within	KTM	0.296	0.298	0.208	0.002	0.720	0.487	0.626

bioRxiv preprint doi: https://doi.org/10.64898/2026.02.17.706398; this version posted February 18, 2026. The copyright holder for this preprint (which was not certified by peer review) is the author/funder, who has granted bioRxiv a license to display the preprint in perpetuity. It is made available under aCC-BY 4.0 International license.

Complexity	FPN	FPN	within	N2O	0.285	0.283	-0.100	0.004	-0.387	0.704	0.756
Complexity	FPN	FPN	within	Sleep N1	0.282	0.283	0.055	0.002	0.313	0.756	0.756
Complexity	FPN	FPN	within	Sleep N2	0.282	0.278	-0.454	0.002	-2.447	0.021	0.039
Complexity	FPN	FPN	within	PPF1.9	0.287	0.276	-0.777	0.004	-2.907	0.012	0.037
Complexity	FPN	FPN	within	PPF2.4	0.174	0.165	-0.989	0.002	-5.043	0.000	0.000
Complexity	FPN	FPN	within	PPF2.7	0.229	0.221	-0.763	0.002	-3.965	0.001	0.002
Complexity	FPN	DMN	between	LSD	0.221	0.231	0.837	0.003	3.241	0.006	0.013
Complexity	FPN	DMN	between	PSIL	0.251	0.261	0.578	0.007	1.530	0.177	0.227
Complexity	FPN	DMN	between	KTM	0.287	0.293	0.505	0.003	1.748	0.108	0.162
Complexity	FPN	DMN	between	N2O	0.275	0.278	0.125	0.005	0.484	0.636	0.715
Complexity	FPN	DMN	between	Sleep N1	0.274	0.274	0.023	0.002	0.132	0.896	0.896
Complexity	FPN	DMN	between	Sleep N2	0.274	0.265	-0.752	0.002	-4.052	0.000	0.001
Complexity	FPN	DMN	between	PPF1.9	0.276	0.266	-0.734	0.004	-2.747	0.017	0.030
Complexity	FPN	DMN	between	PPF2.4	0.162	0.153	-0.879	0.002	-4.480	0.000	0.001
Complexity	FPN	DMN	between	PPF2.7	0.218	0.208	-0.913	0.002	-4.744	0.000	0.001
Complexity	DMN	DMN	within	LSD	0.210	0.216	0.567	0.003	2.196	0.045	0.082
Complexity	DMN	DMN	within	PSIL	0.230	0.234	0.388	0.004	1.025	0.345	0.443
Complexity	DMN	DMN	within	KTM	0.260	0.263	0.253	0.003	0.875	0.400	0.450
Complexity	DMN	DMN	within	N2O	0.253	0.249	-0.296	0.004	-1.148	0.270	0.405
Complexity	DMN	DMN	within	Sleep N1	0.249	0.248	-0.077	0.001	-0.444	0.660	0.660
Complexity	DMN	DMN	within	Sleep N2	0.249	0.241	-0.724	0.002	-3.897	0.001	0.002
Complexity	DMN	DMN	within	PPF1.9	0.252	0.241	-0.880	0.003	-3.293	0.006	0.013
Complexity	DMN	DMN	within	PPF2.4	0.153	0.143	-1.238	0.002	-6.315	0.000	0.000
Complexity	DMN	DMN	within	PPF2.7	0.203	0.194	-1.106	0.002	-5.746	0.000	0.000

Abbreviations: FC: functional connectivity; LSD: lysergic acid diethylamide; PSIL: psilocybin; KTM: ketamine; N_2O: nitrous oxide; PPF1.9 / PPF2.4 / PPF2.7: propofol (effect-site concentrations: 1.9 / 2.4 / 2.7 $\mu g \cdot mL^{-1}$). U-U: unimodal-unimodal; A-A: attention-attention; T-T: transmodal-transmodal; U-A: unimodal-attention; U-T: unimodal-transmodal; A-T: attention-transmodal. VIS: visual network; SMN: somatomotor network; DAN: dorsal attention network; VAN: ventral attention network; LIM: limbic network; FPN: frontoparietal network; DMN: default-mode network.
